# Supplementary material for: Positive modulators of N-methyl-d-aspartate receptor: structure–activity relationship study on steroidal C-17 and C-20 oxime ethers
Source: RSC Adv. 2026 Feb 24;16(12):10769–84. doi: 10.1039/d5ra07716h (PMC12930396; doi:10.1039/d5ra07716h)

Supplementary Information (SI) for RSC Advances.
This journal is © The Royal Society of Chemistry 2026

Title: Positive Modulators of *N*-Methyl-*D*-Aspartate Receptor: Structure-Activity Relationship Study on Steroidal C-17 and C-20 Oxime Ethers

Authors:
Santosh Kumar Adla,[a] Barbora Hrcka Krausová,[b] Bohdan Kysilov,[b] Karel Kudláček,[a] Radko Souček,[a] Miloš Budešínský,[a] Jan Voldřich,[a,c] Ladislav Vyklický,[b] Eva Kudová [a,*]

Supporting information

[a]Institute of Organic Chemistry and Biochemistry of the Czech Academy of Sciences, Flemingovo nam. 2, Prague 6 – Dejvice, 16610, Czech Republic
[b]Institute of Physiology of the Czech Academy of Sciences, Videnska 1083, Prague 4, 14220, Czech Republic
[c]University of Chemistry and Technology, Technická 5, Prague 6 – Dejvice, 166 28 Czech Republic

Table of Contents

^{1}H and ^{13}C NMR spectra of compounds 11-24 2
HR-MS and LC-MS spectra of compounds 11-28 11

^{1}H and ^{13}C NMR spectra of compounds **11-24**

Figure S1. ^{1}H NMR and ^{13}C NMR and spectra of compound **11**

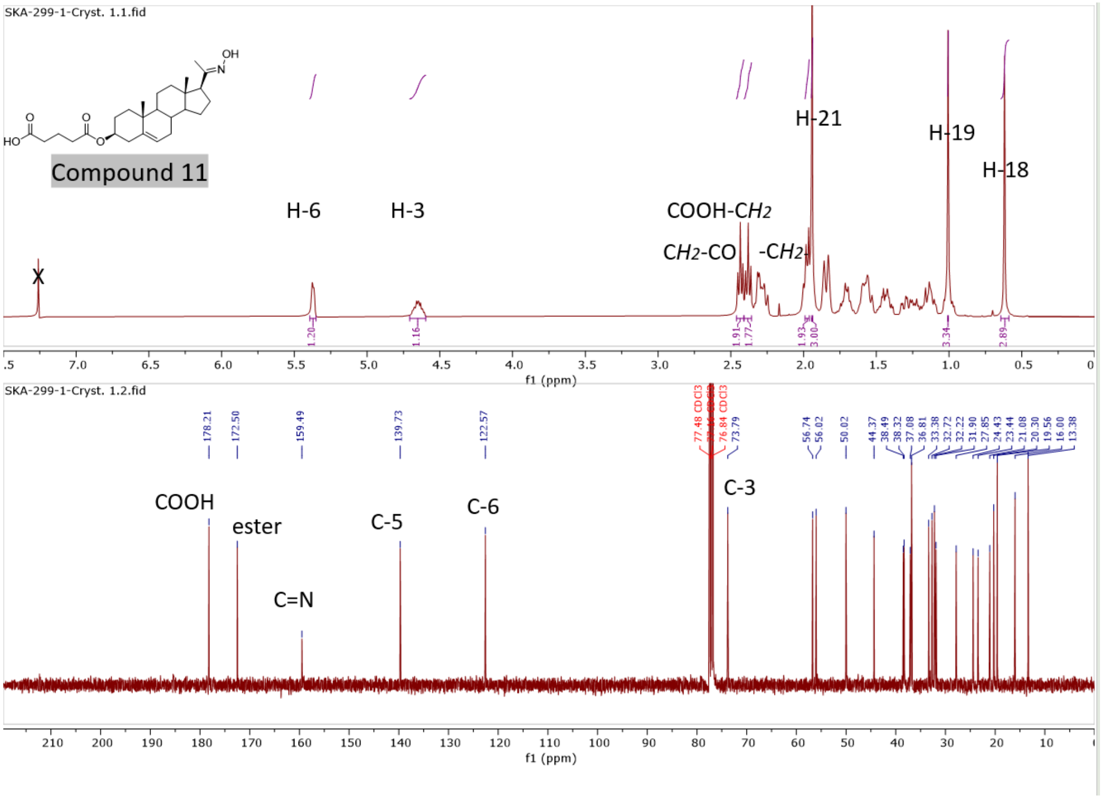

Figure S2. ^{1}H NMR and ^{13}C NMR and spectra of compound **12**

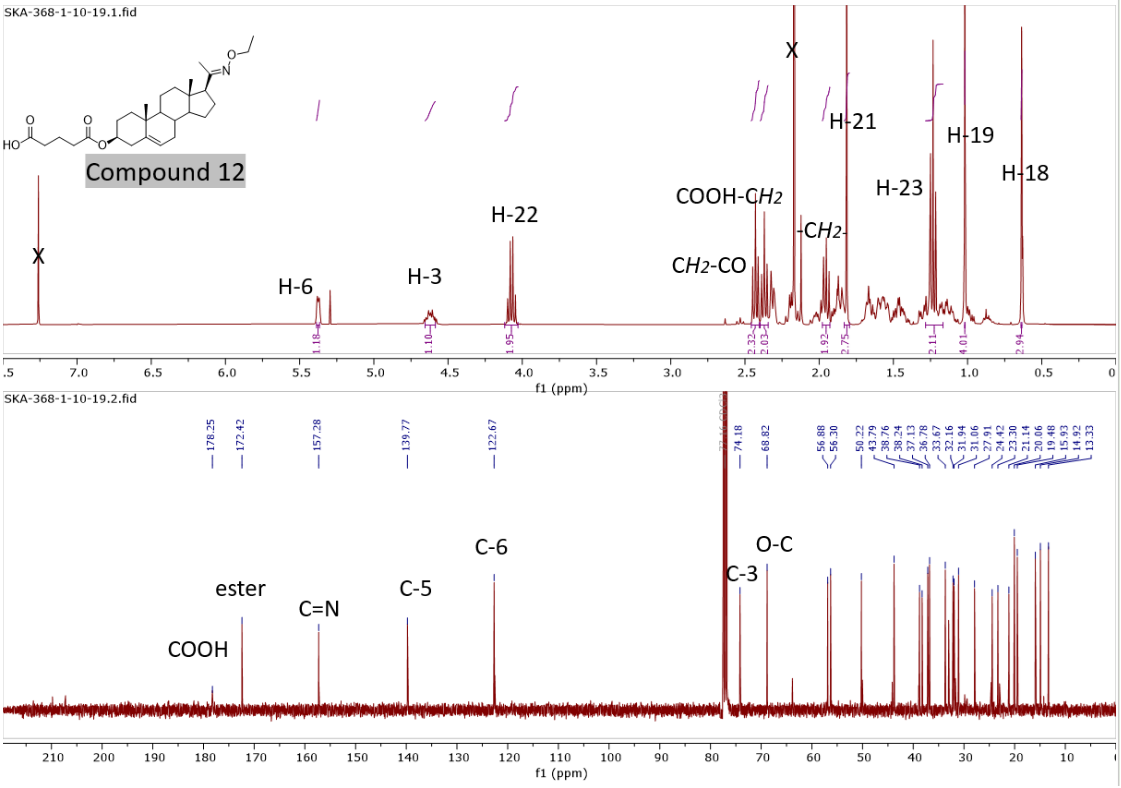

Figure S3. ^{1}H NMR and ^{13}C NMR and spectra of compound **13**

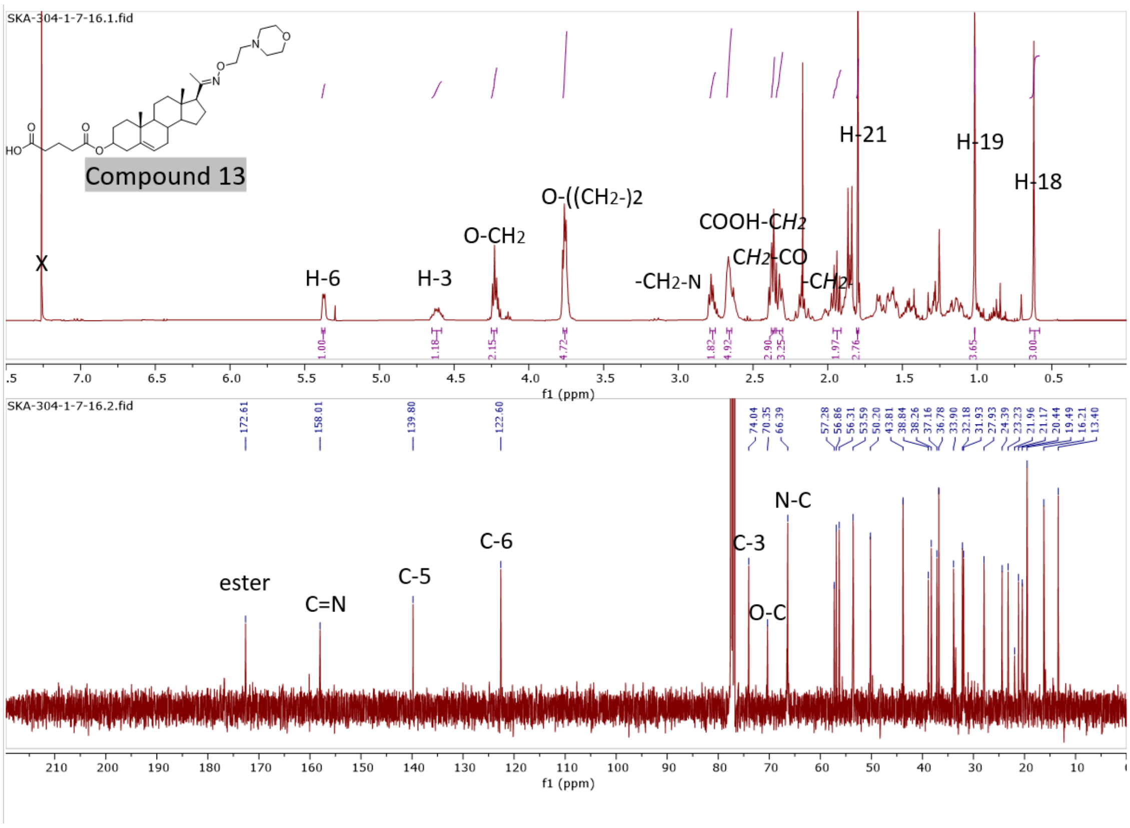

Figure S4. ^{1}H NMR and ^{13}C NMR and spectra of compound **14**

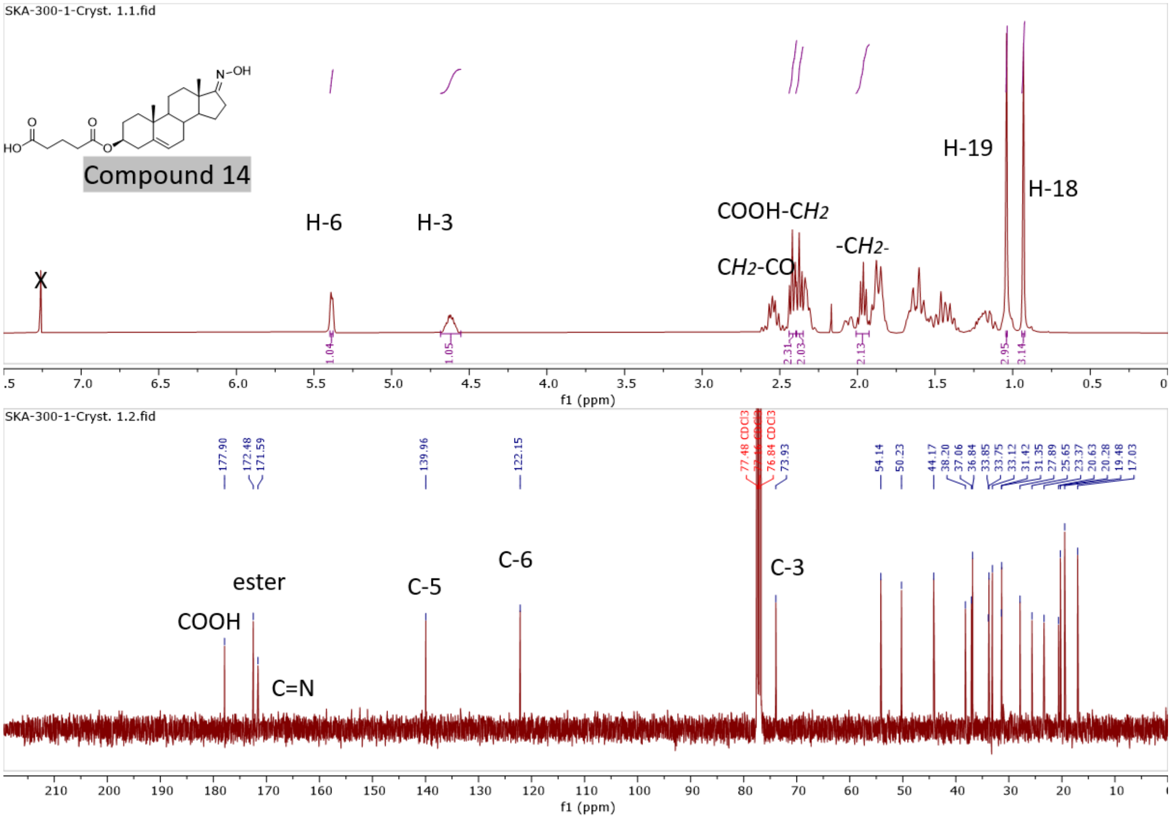

Figure S5. ^{1}H NMR and ^{13}C NMR and spectra of compound **15**

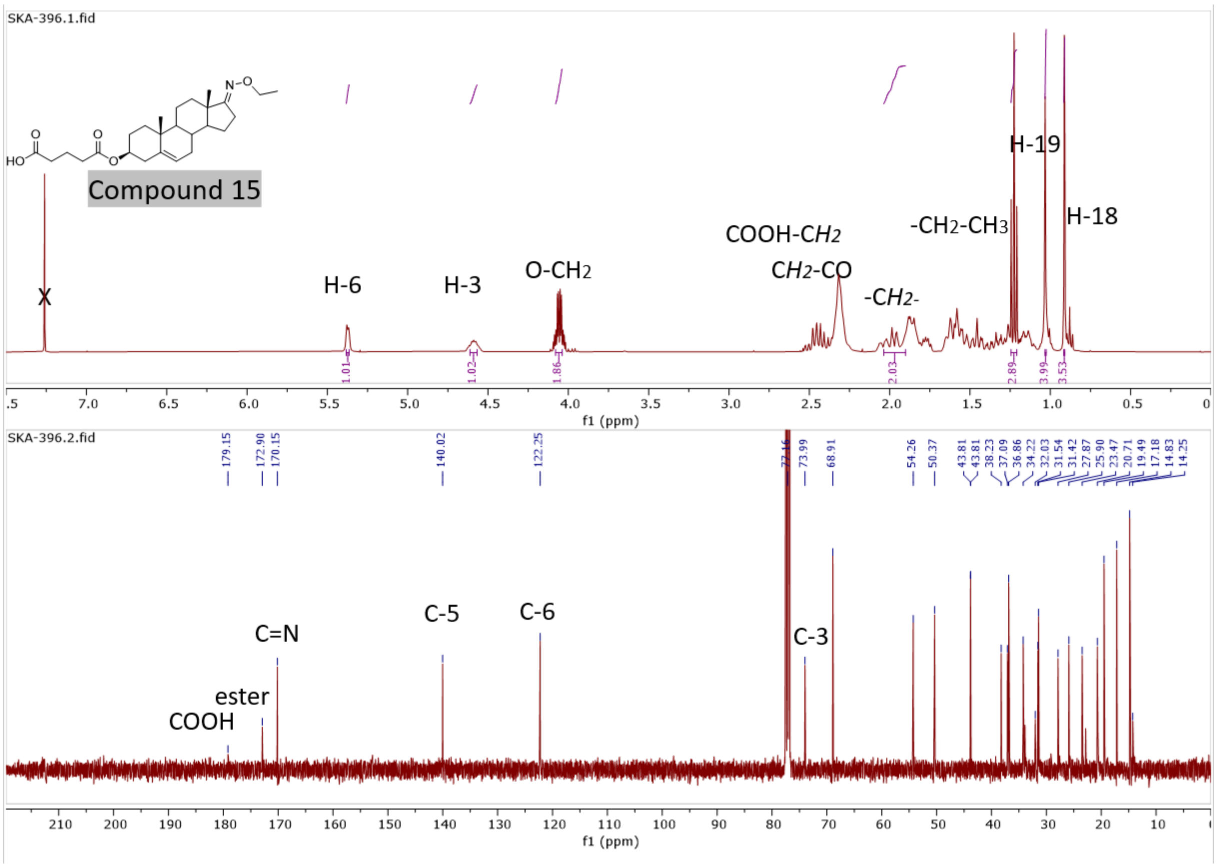

Figure S6. ^{1}H NMR and ^{13}C NMR and spectra of compound **16**

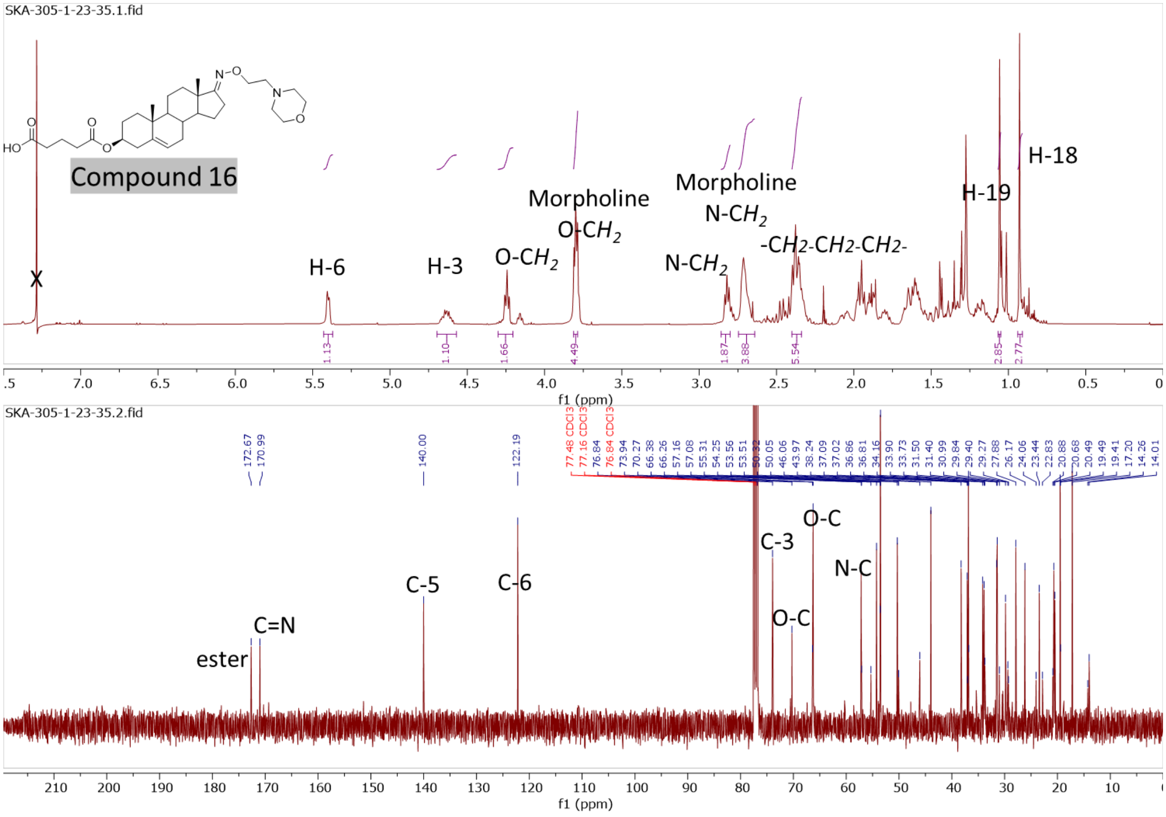

Figure S7. ^{1}H NMR and ^{13}C NMR and spectra of compound **17**

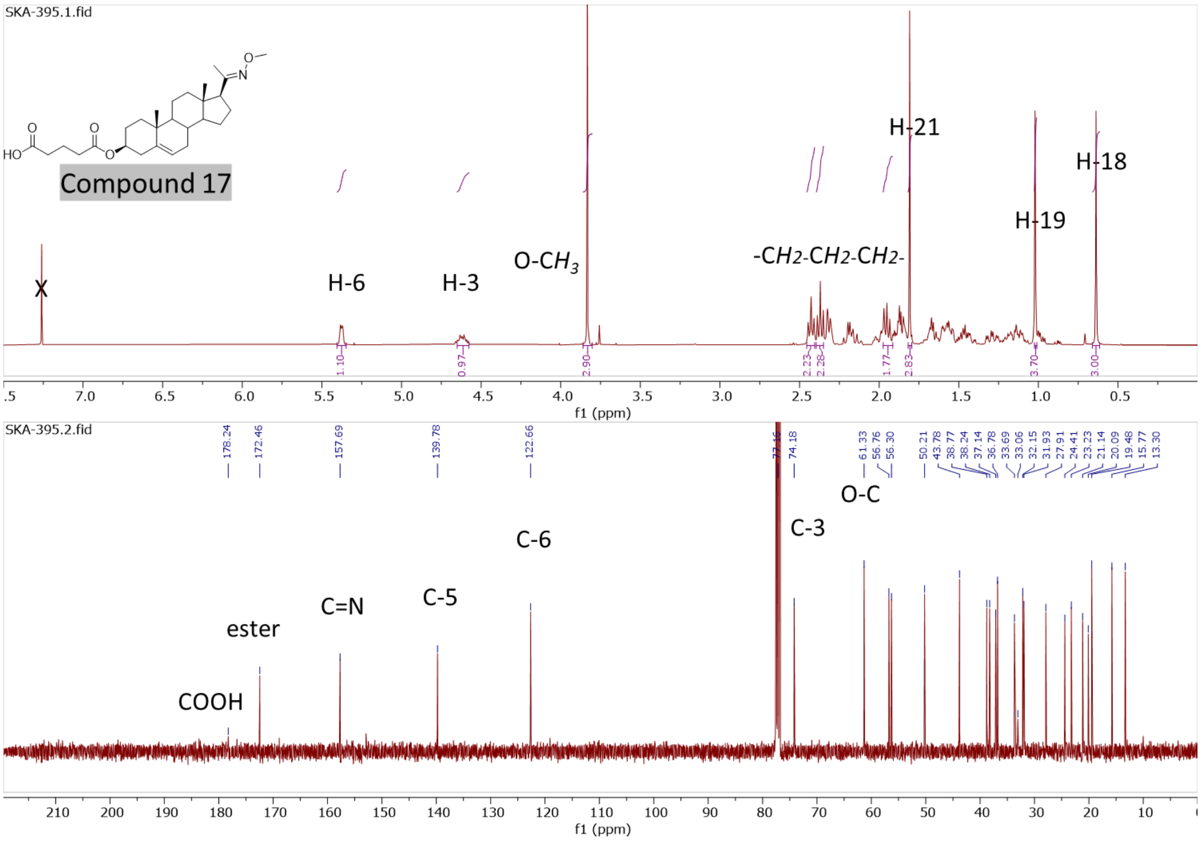

Figure S8. ^{1}H NMR and ^{13}C NMR and spectra of compound **18**

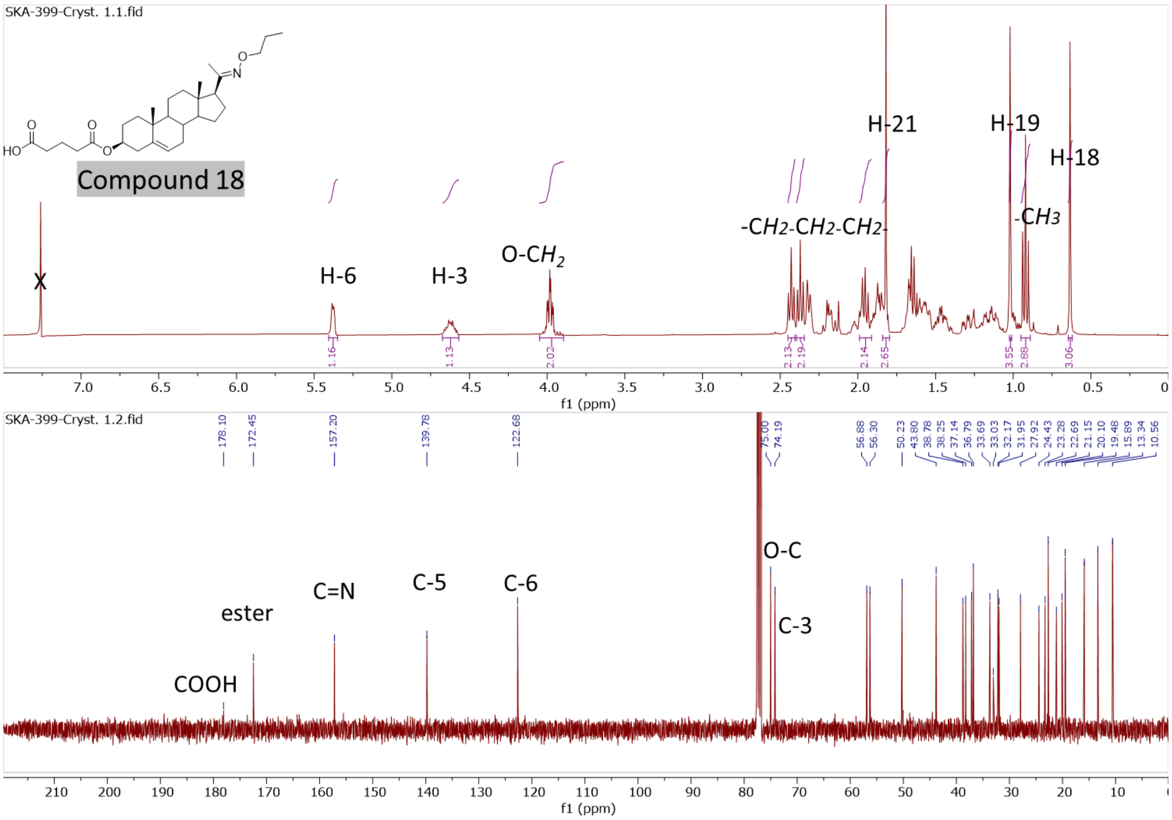

Figure S9. ^{1}H NMR and ^{13}C NMR and spectra of compound **19**

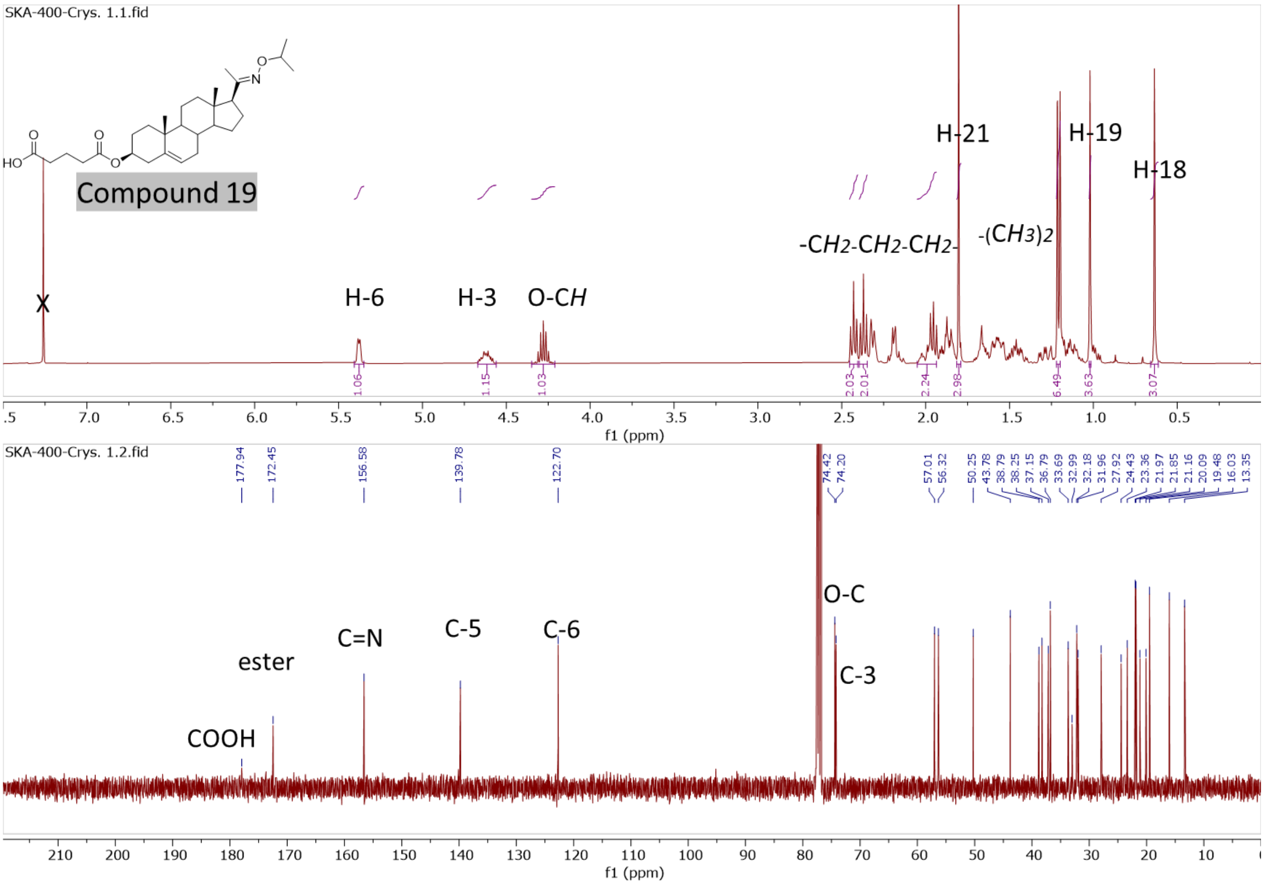

Figure S10. ^{1}H NMR and ^{13}C NMR and spectra of compound **20**

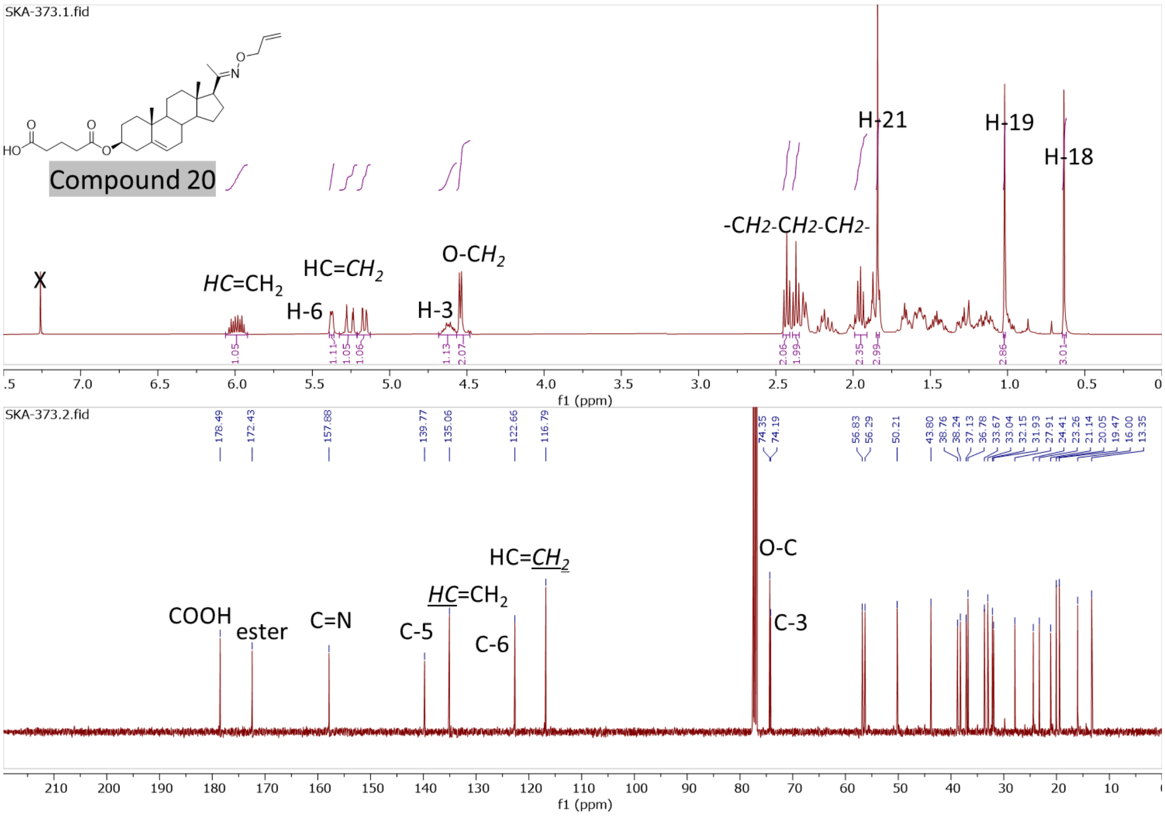

Figure S11. ^{1}H NMR and ^{13}C NMR and spectra of compound **21**

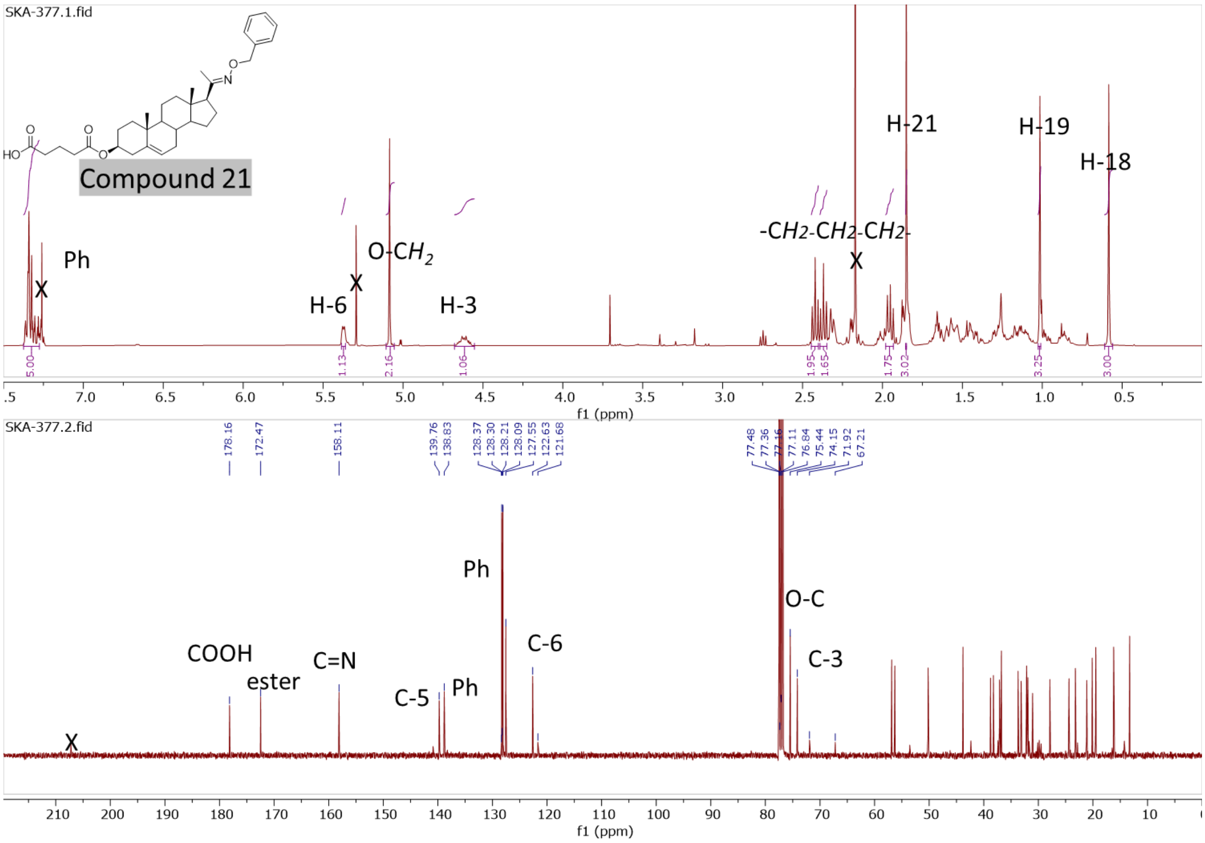

Figure S12. ^{1}H NMR and ^{13}C NMR and spectra of compound **22**

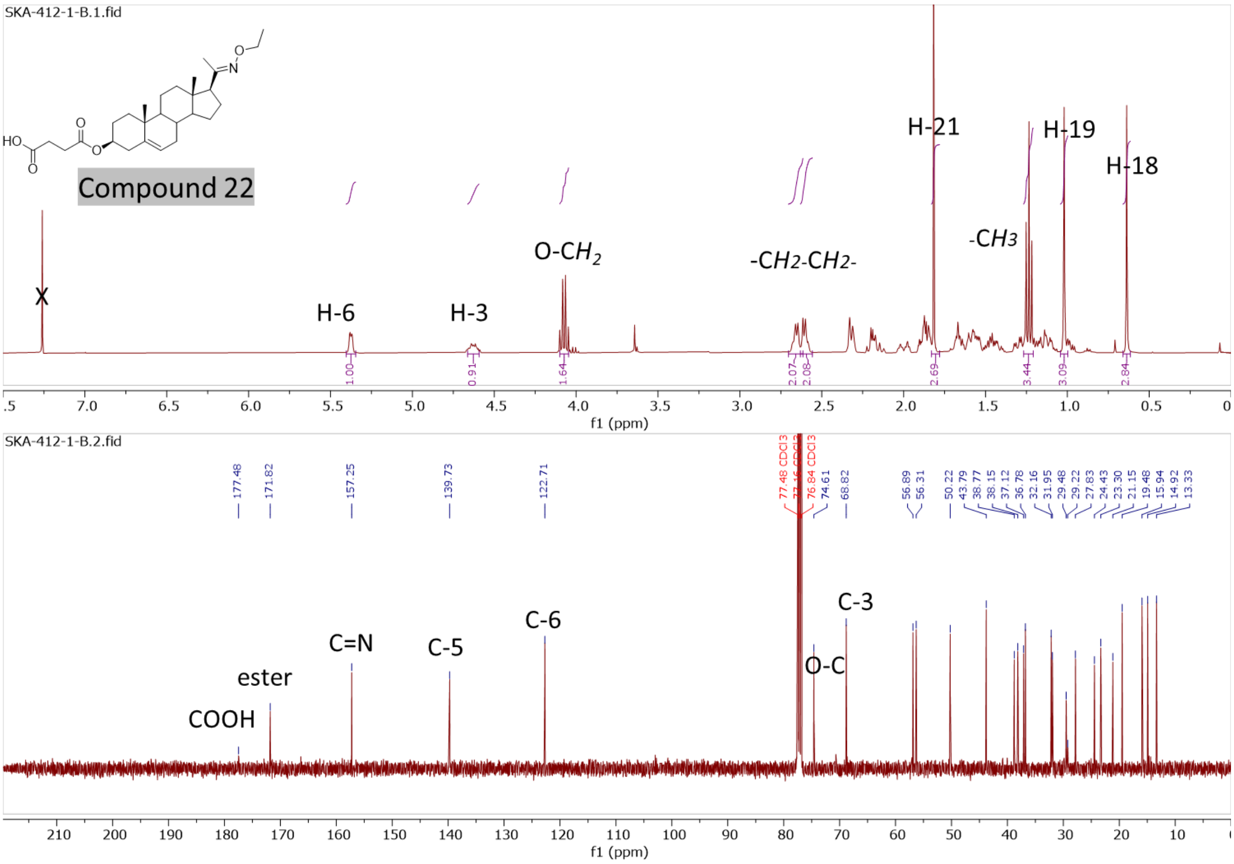

Figure S13. ^{1}H NMR and ^{13}C NMR and spectra of compound **23**

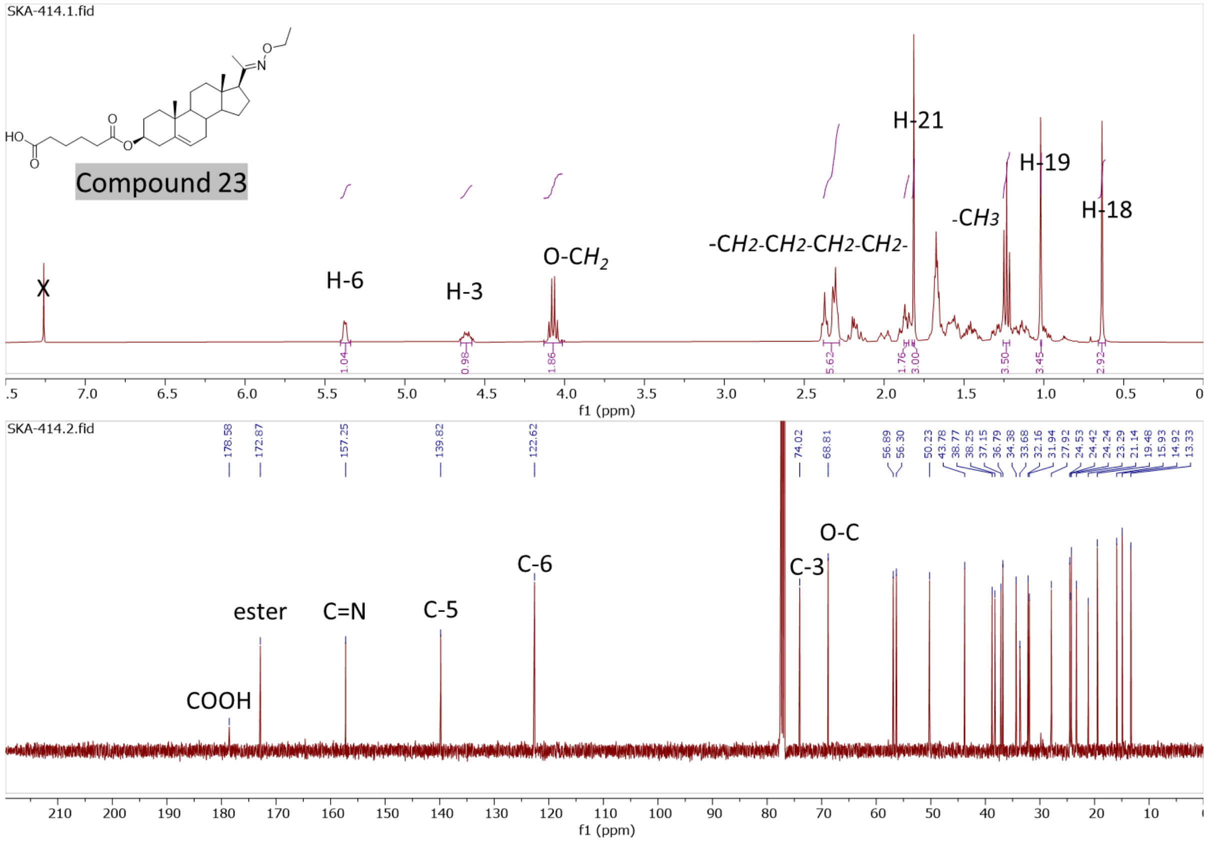

Figure S14. ^{1}H NMR and ^{13}C NMR and spectra of compound **24**

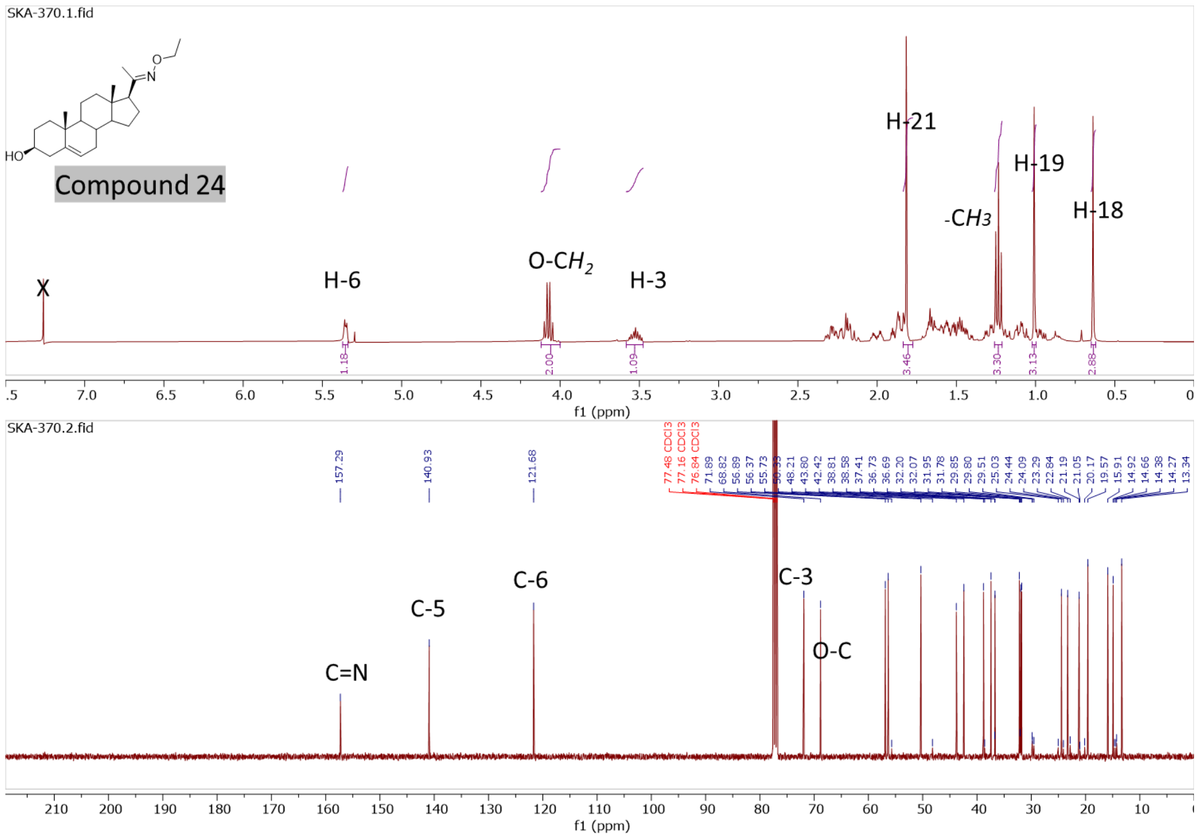

Figure S15. ^{1}H NMR and ^{13}C NMR and spectra of compound **25**

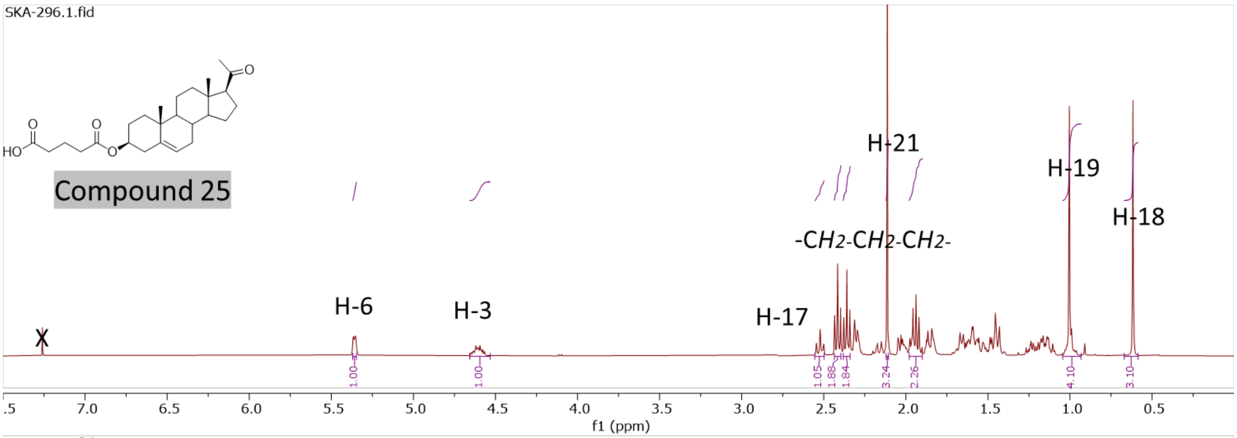

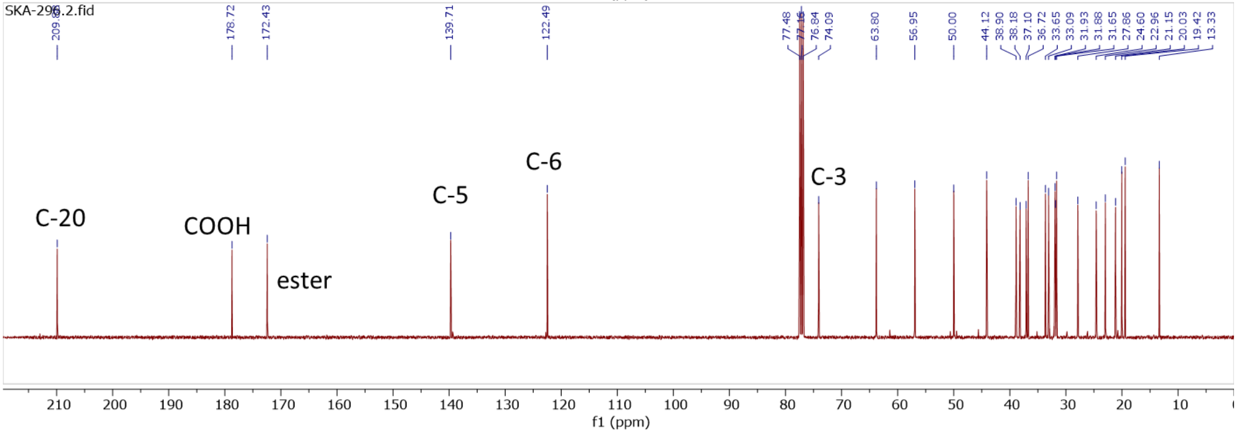

Figure S16. ^{1}H NMR and ^{13}C NMR and spectra of compound **26**

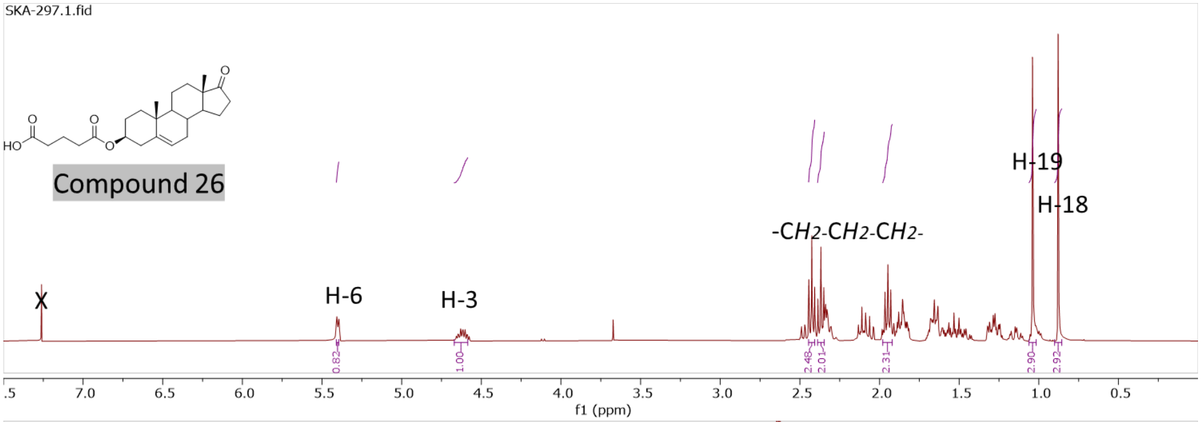

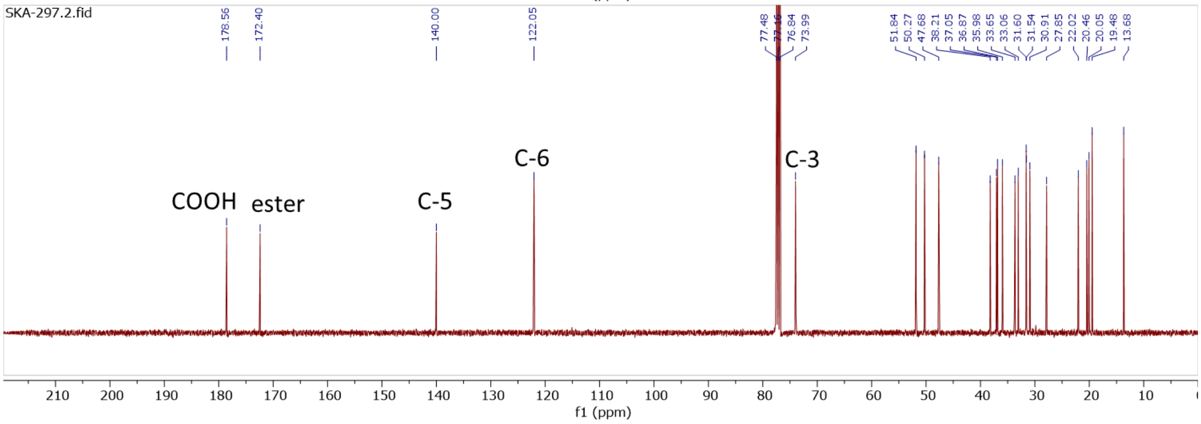

Figure S17. ^{1}H NMR and ^{13}C NMR and spectra of compound **27**

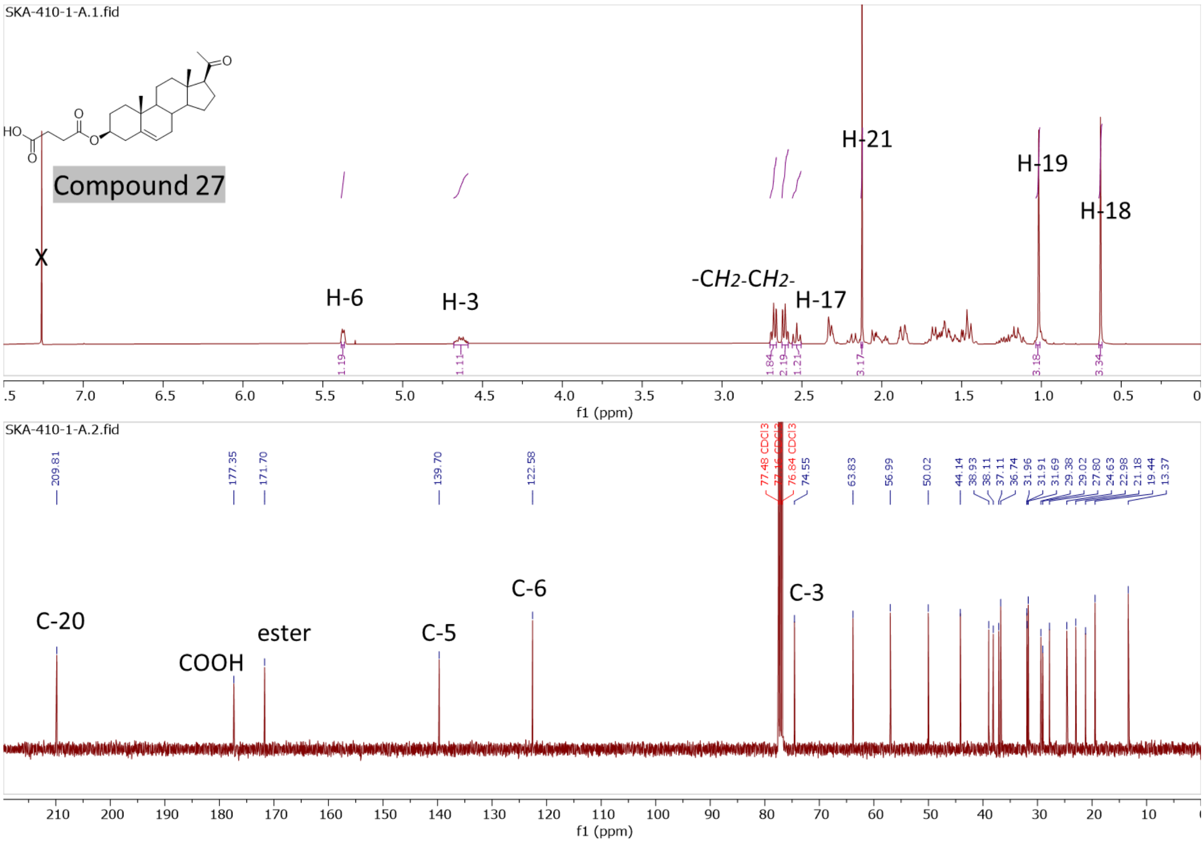

Figure S18. ^{1}H NMR and ^{13}C NMR and spectra of compound **28**

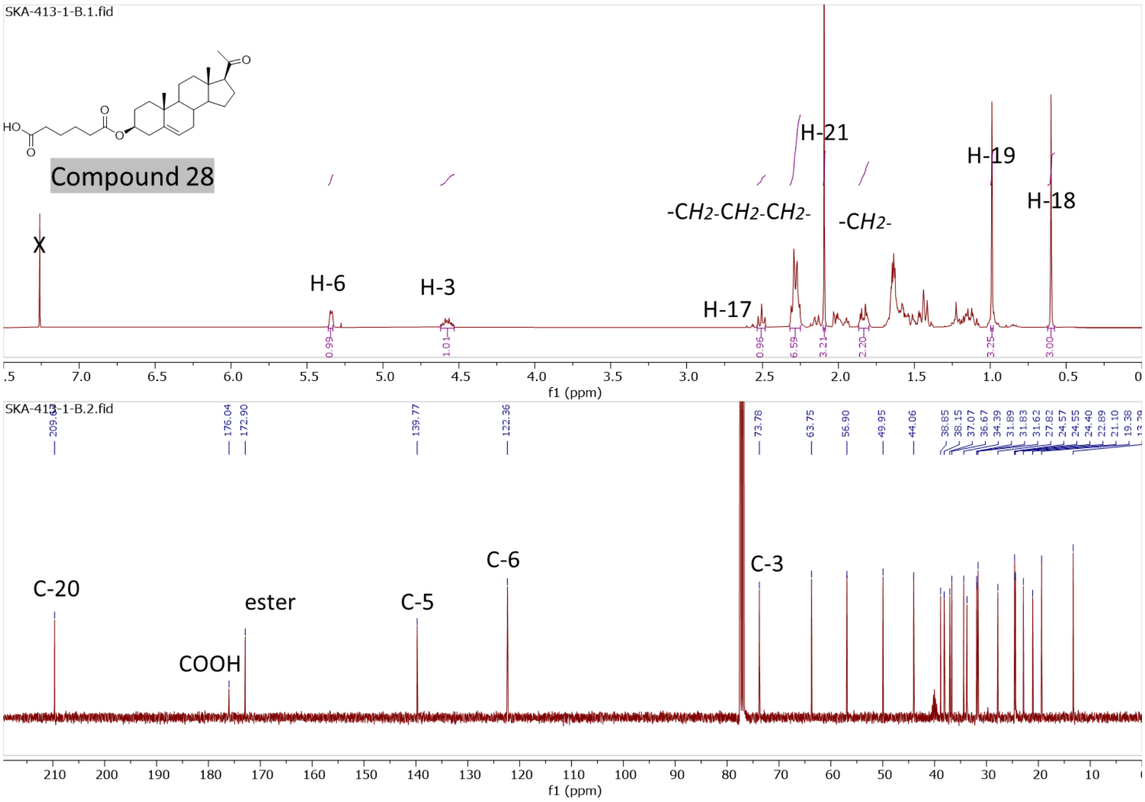

HR-MS and LC-MS spectra of compounds **11**-**28**

Figure S19. HR-MS and LC-MS spectra of compound **11**

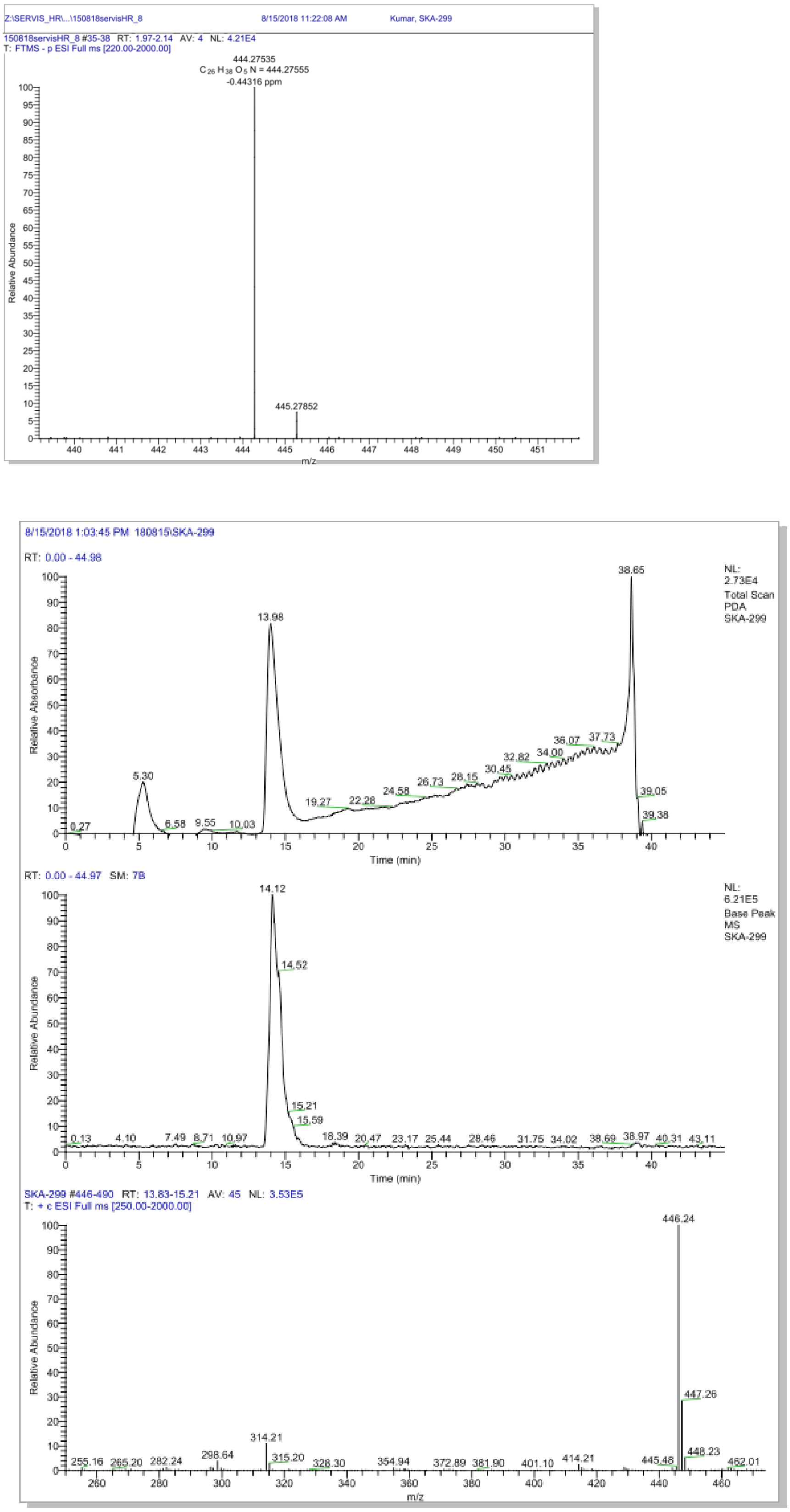

Figure S20. HR-MS and LC-MS spectra of compound **12**

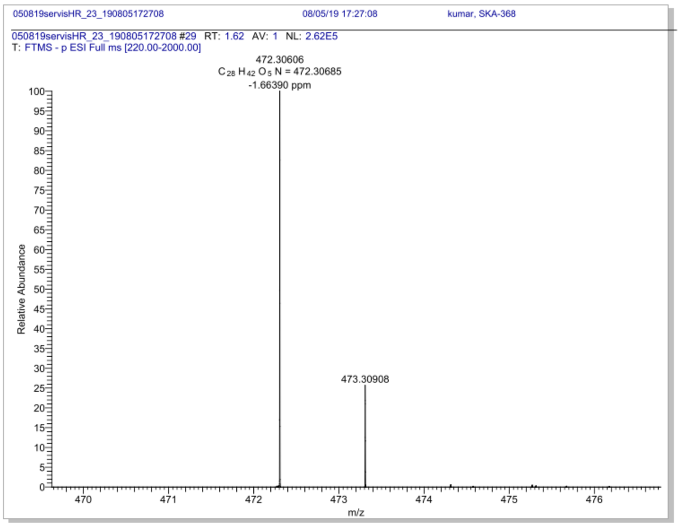

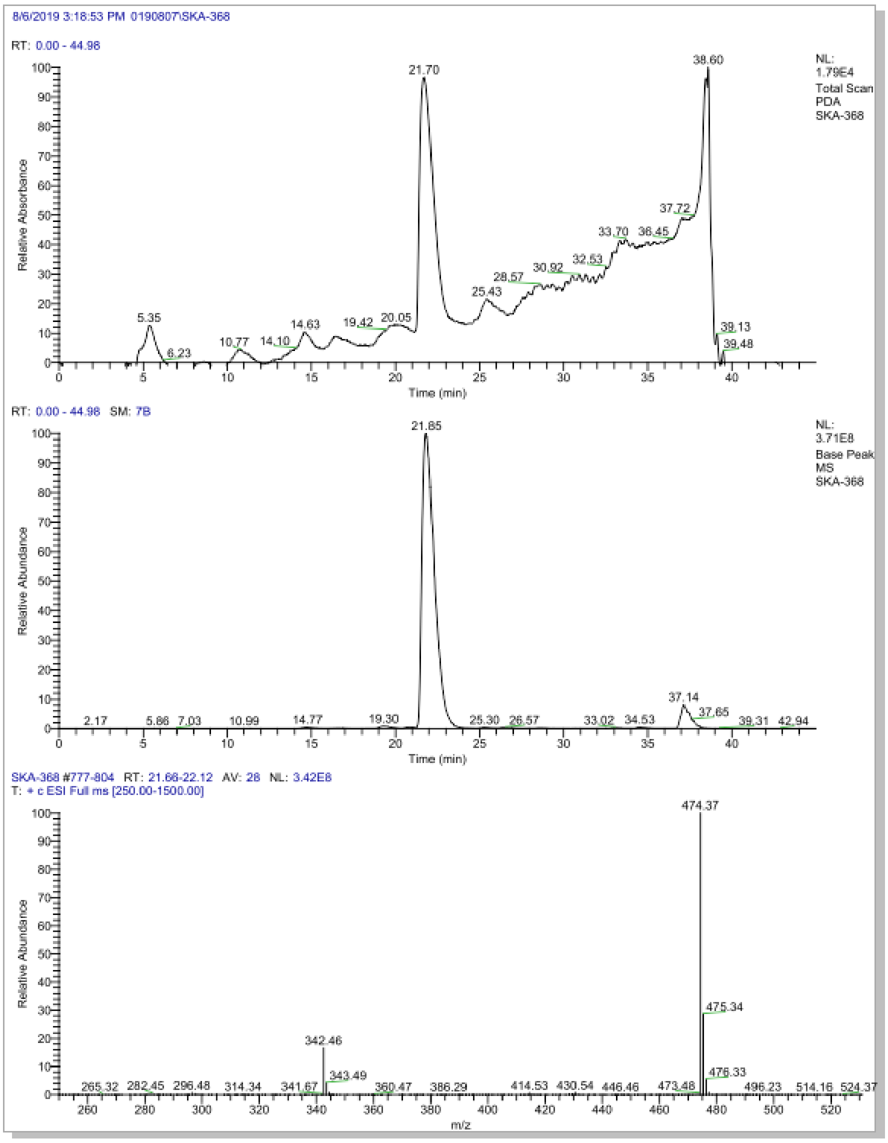

Figure S21. HR-MS and LC-MS spectra of compound **13**

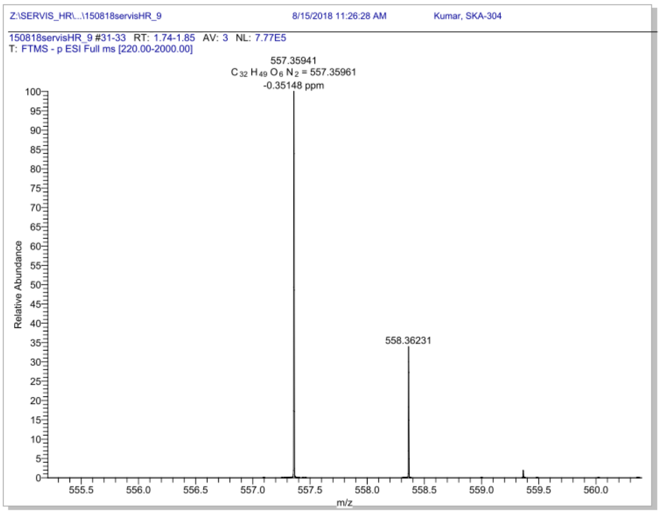

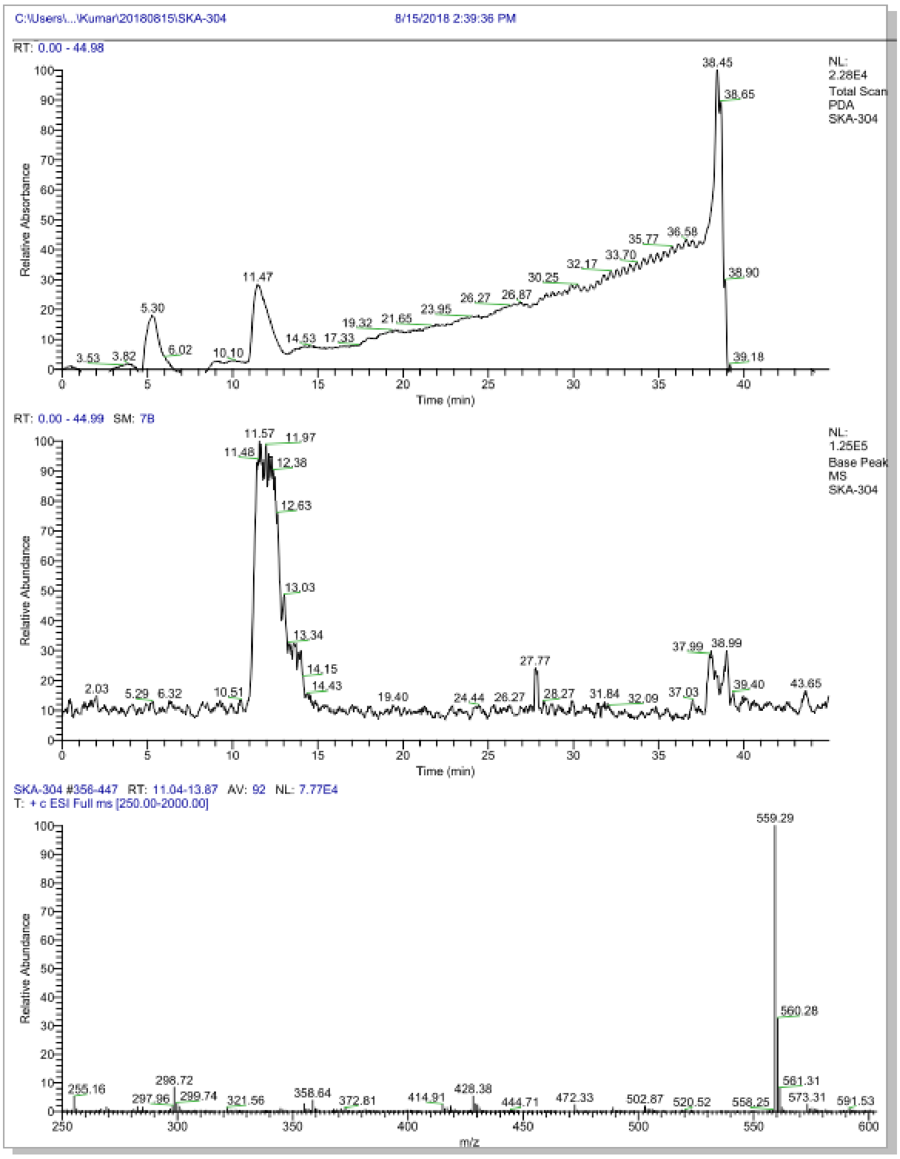

Figure S22. HR-MS and LC-MS spectra of compound **14**

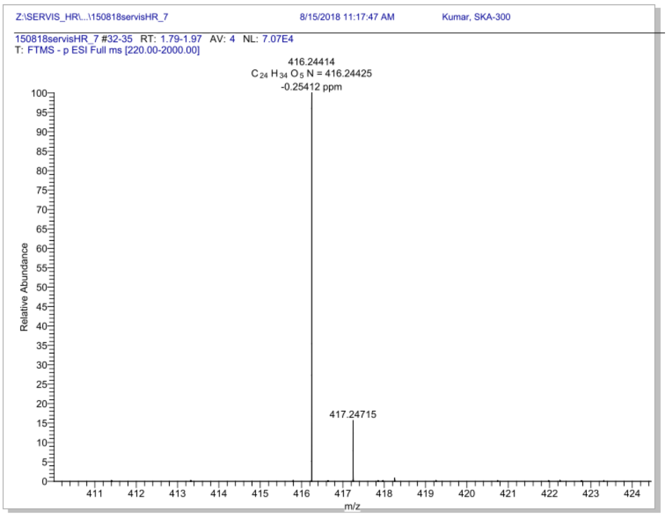

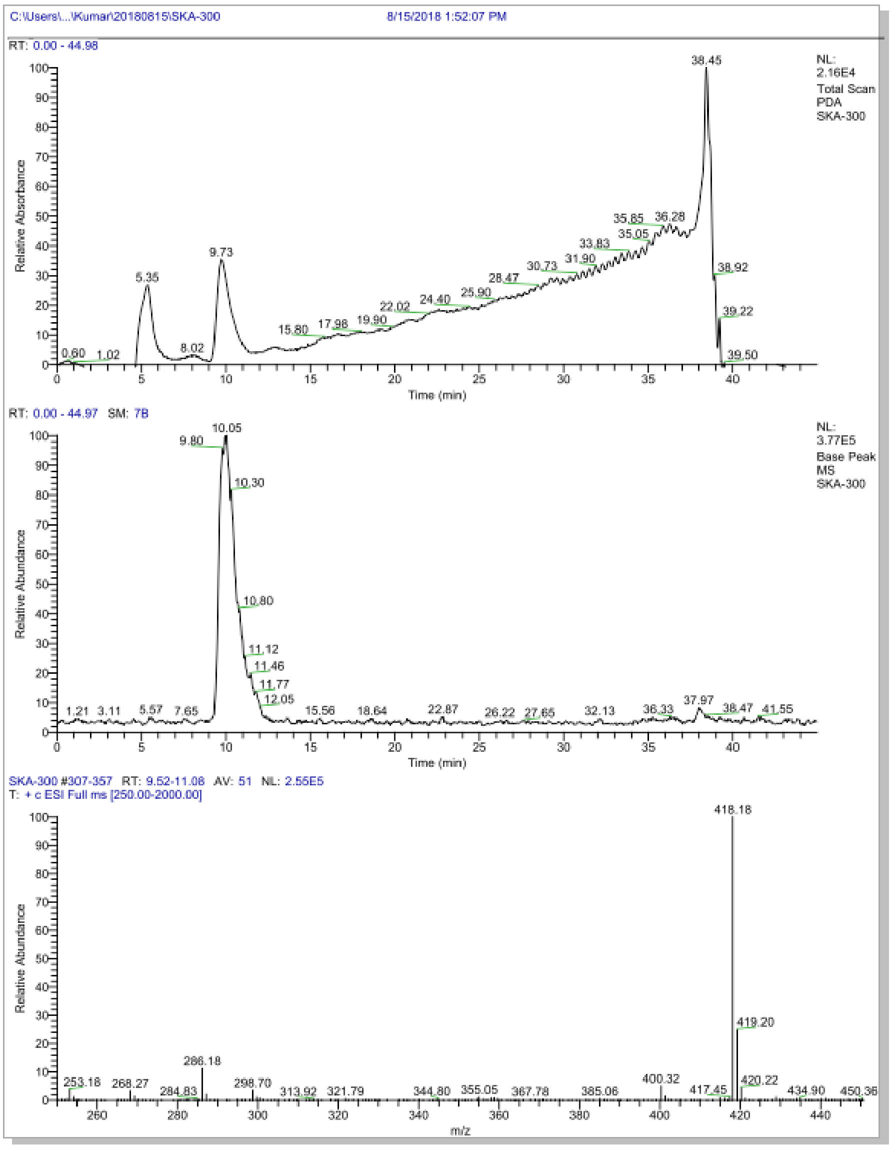

Figure S23. HR-MS and LC-MS spectra of compound **15**

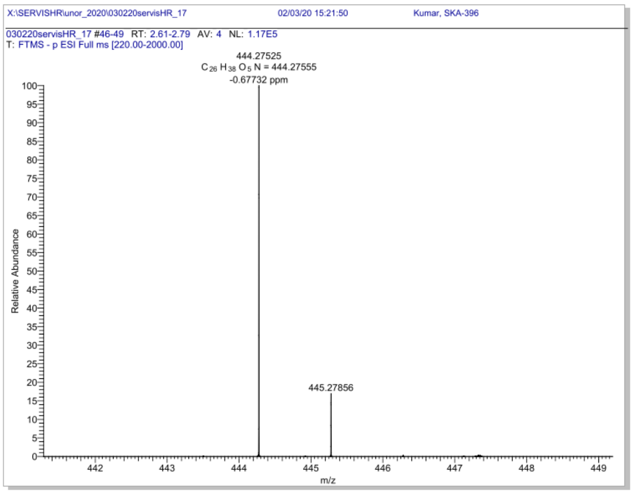

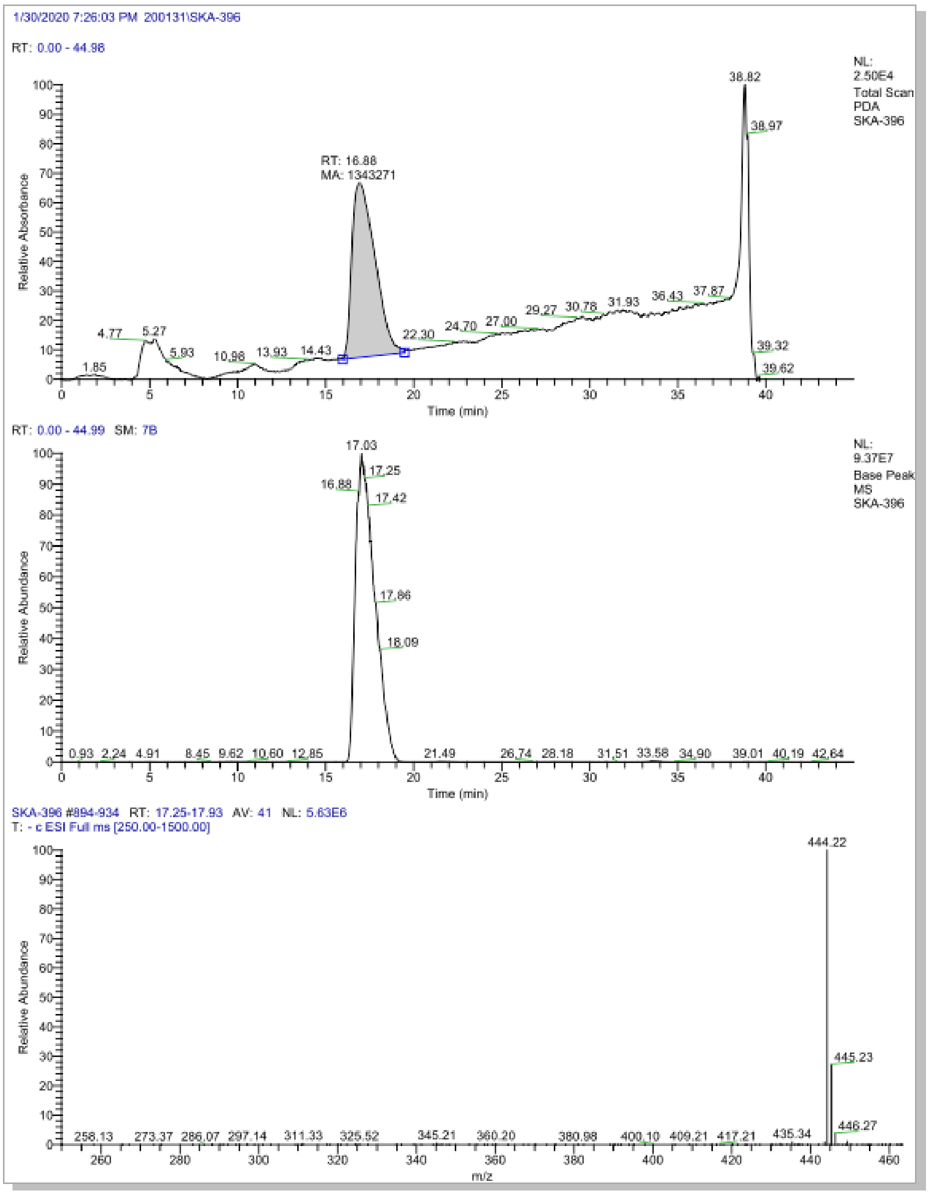

Figure S24. HR-MS and LC-MS spectra of compound **16**

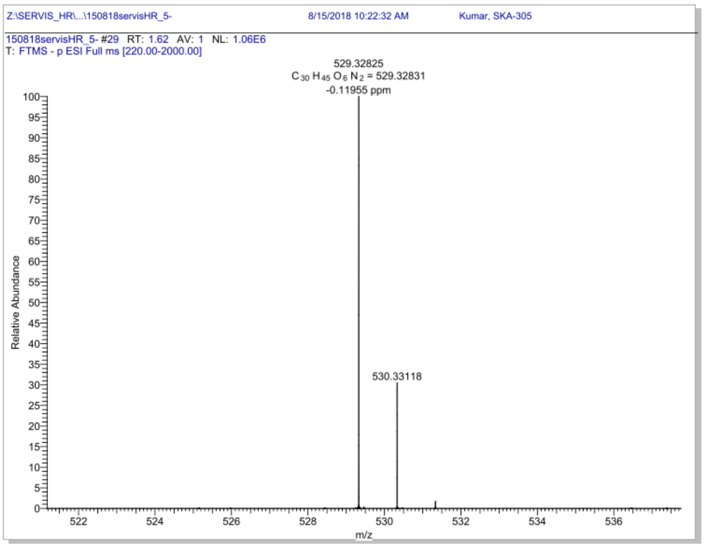

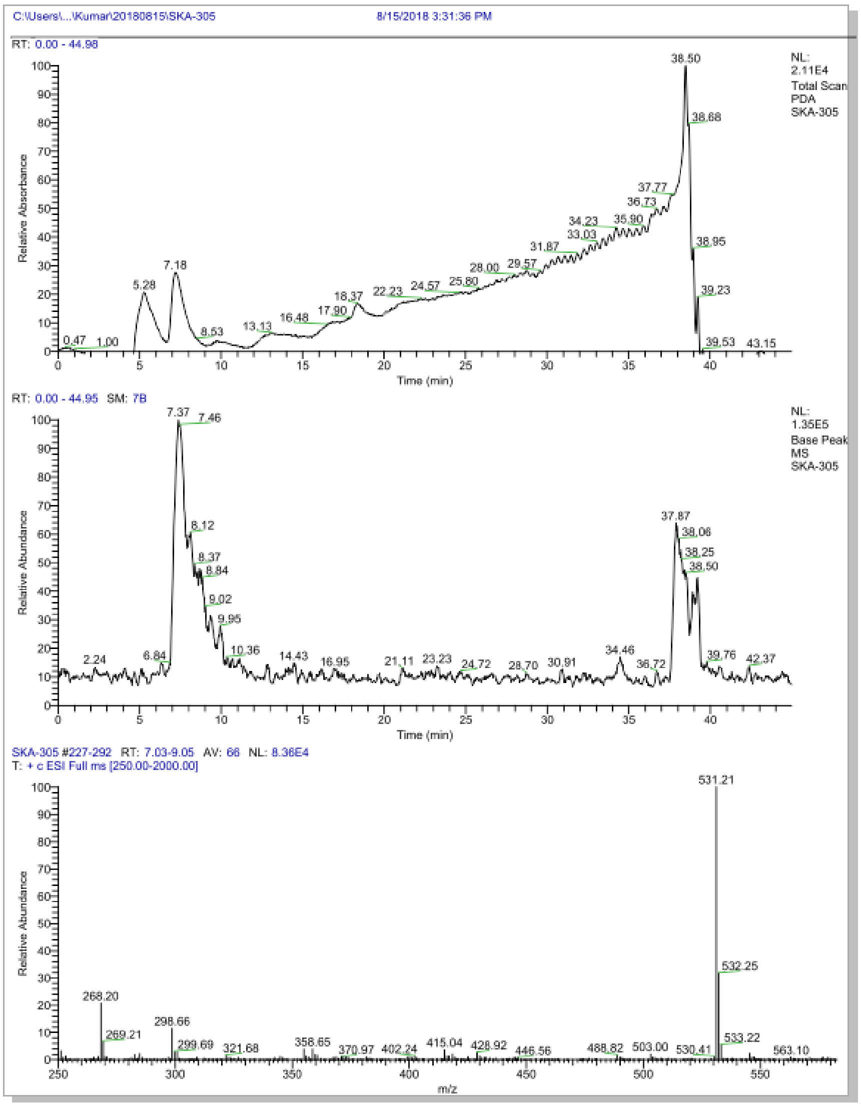

Figure S25. HR-MS and LC-MS spectra of compound **17**

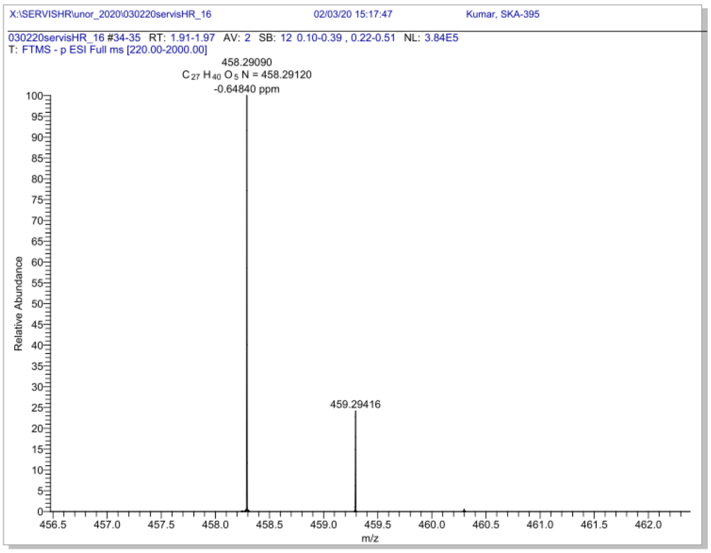

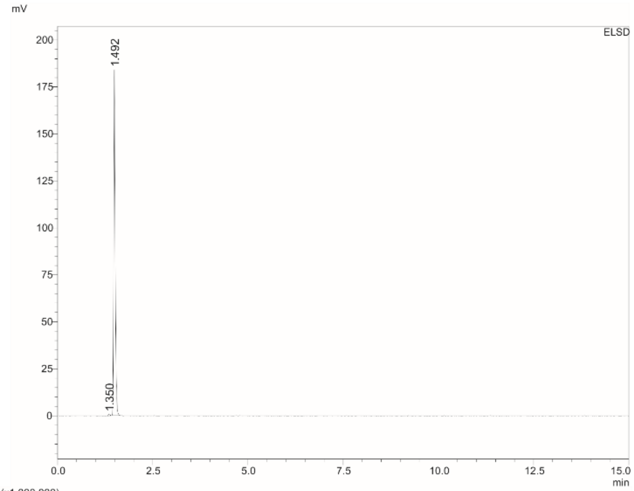

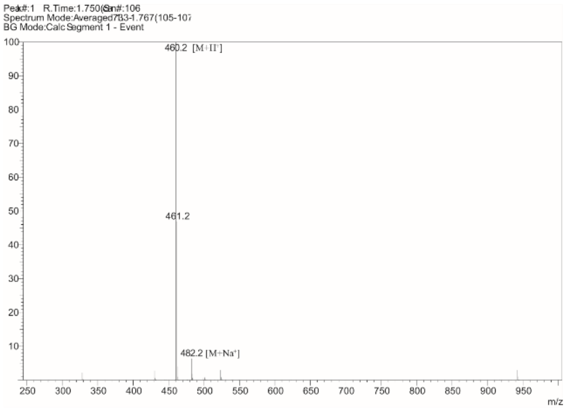

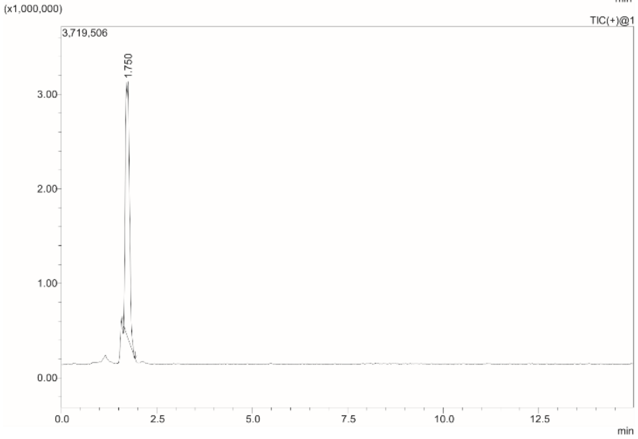

Box :
Position :
Method Filename : C8-80,20.lcm
Vial # : 1-2
Injection Volume : 0.3 uL
Date Acquired : 25-Aug-22 12:30:51

Comment : Isocratic, A: H2O, MeOH, FA (950:50:1); B: ACN; A:B (80:20), 0.5 mL/min
Column, Shim-pack Scepter, C8-120, 1.9 um, 2.1x100 mm, (Shimadzu)

ELSD

Ret. Time	Area	Area%
1.350	2255	0.410
1.492	548194	99.590
	550450	100.000

Figure S26. HR-MS and LC-MS spectra of compound **18**

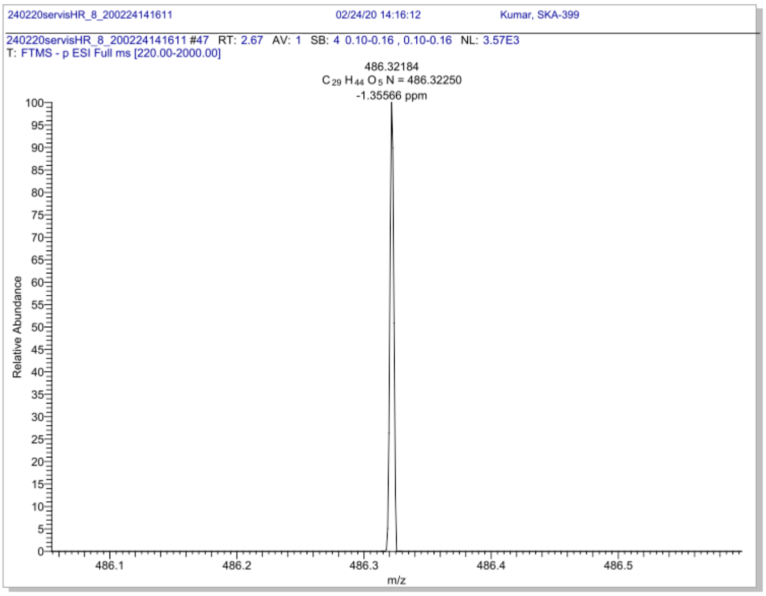

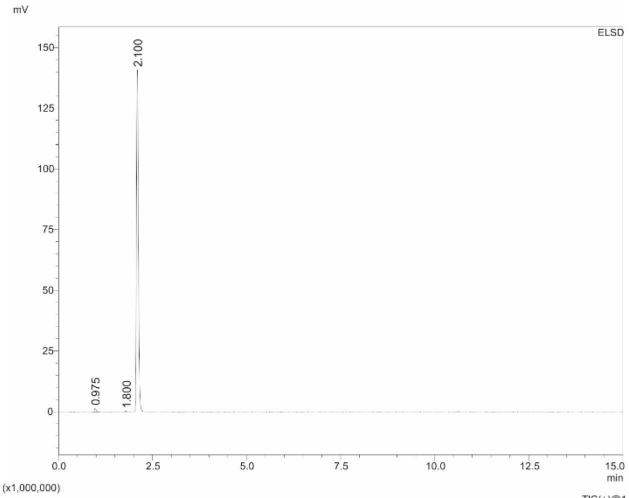

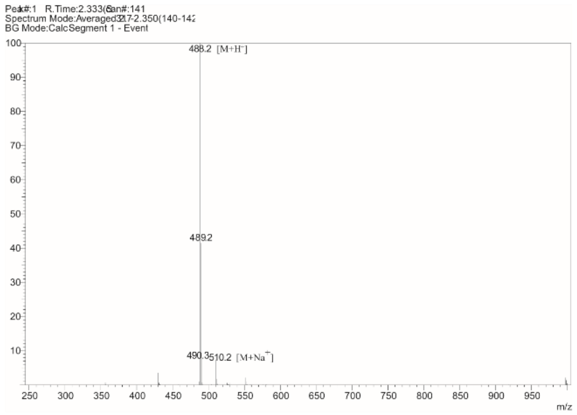

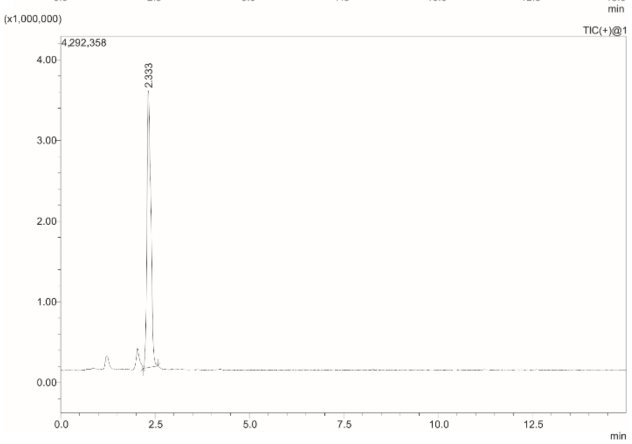

Box :
Position :
Method Filename : C8-80,20.lcm
Vial # : 1-3
Injection Volume : 0.1 uL
Date Acquired : 25-Aug-22 13:16:04

Comment : Isocratic, A: H2O, MeOH, FA (950:50:1); B: ACN; A:B (80:20), 0.5 mL/min
Column, Shim-pack Scepter, C8-120, 1.9 um, 2.1x100 mm, (Shimadzu)

ELSD

Ret. Time	Area	Area%
0.975	4439	0.918
1.800	868	0.179
2.100	478375	98.903
	483681	100.000

Figure S27. HR-MS and LC-MS spectra of compound **19**

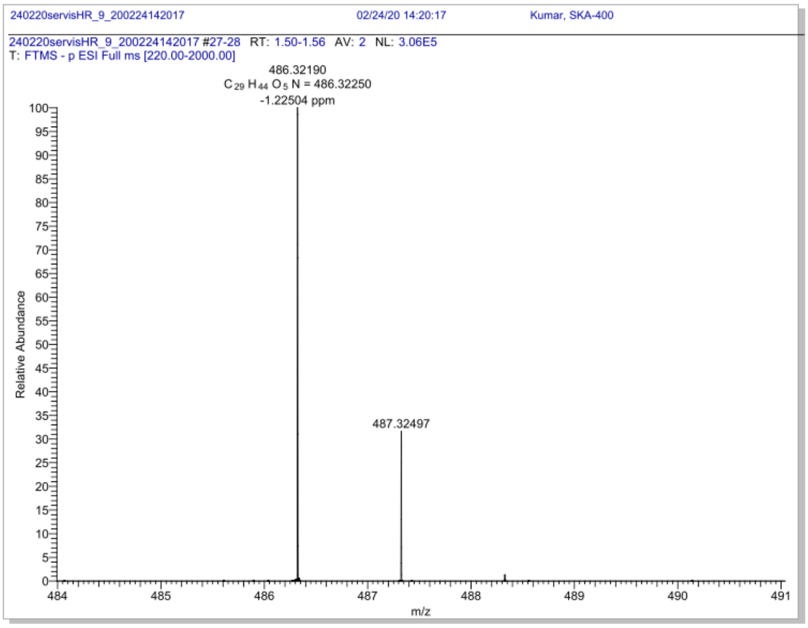

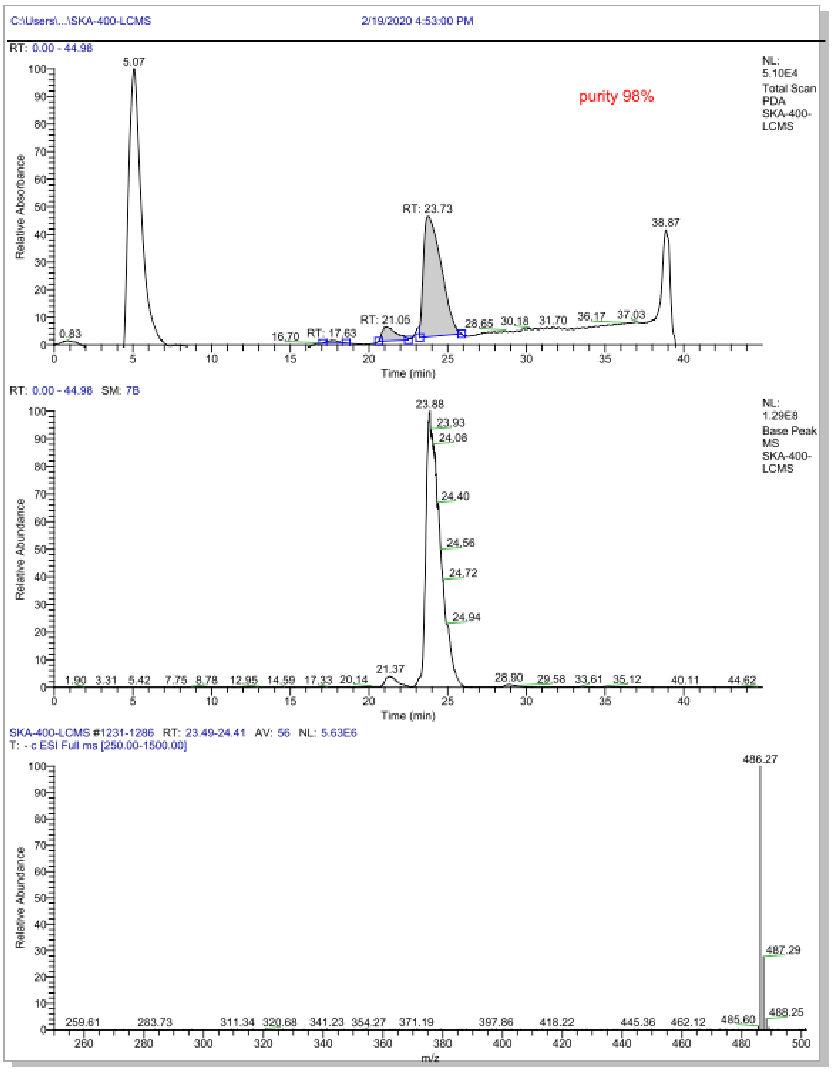

Figure S28. HR-MS and LC-MS spectra of compound **20**

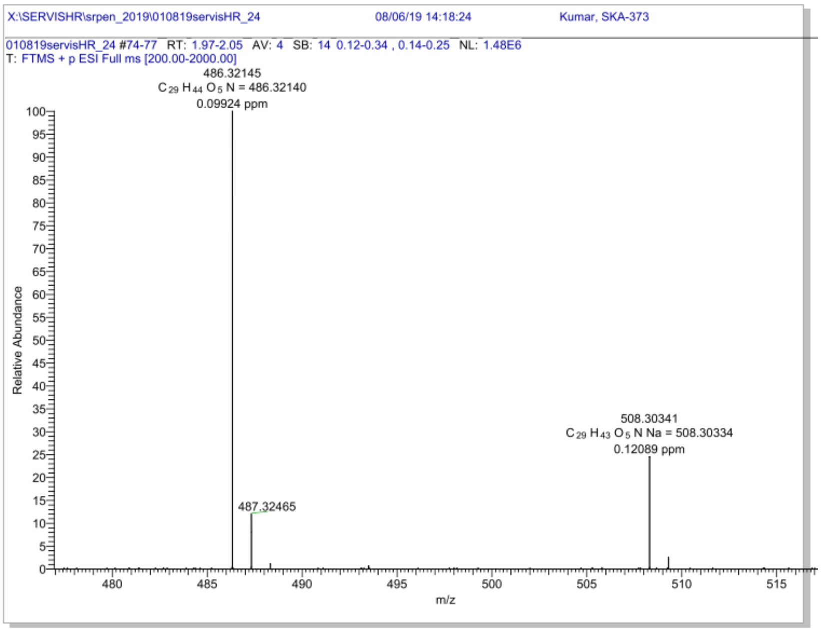

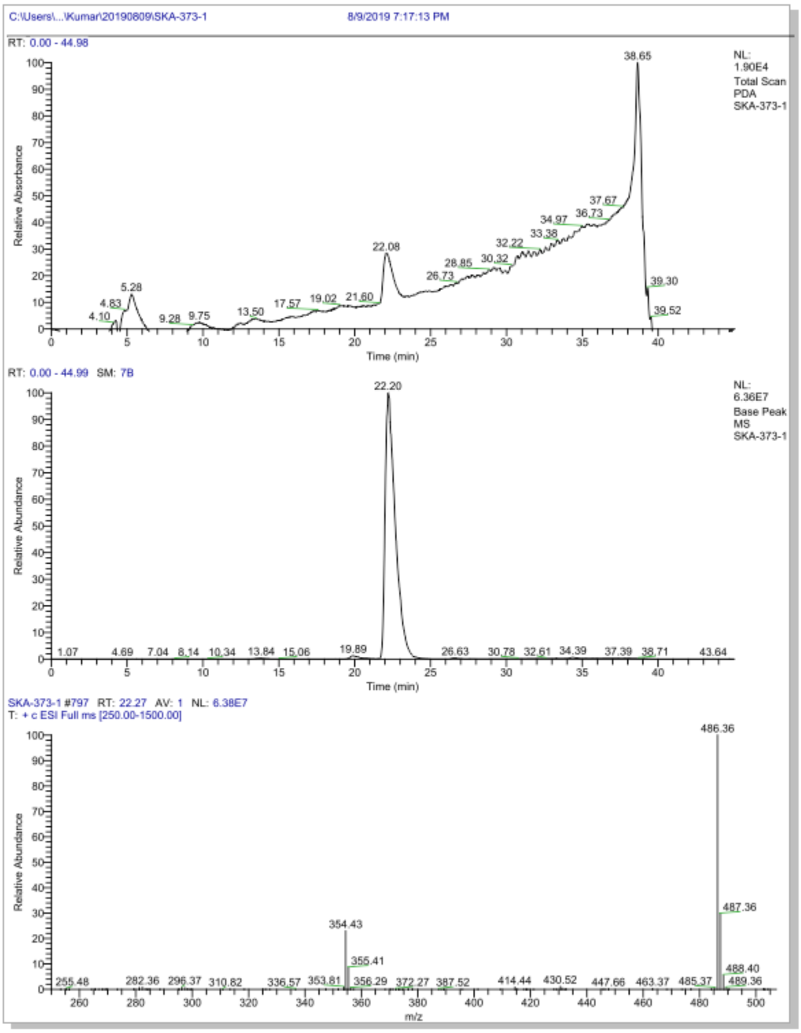

Figure S29. HR-MS and LC-MS spectra of compound **21**

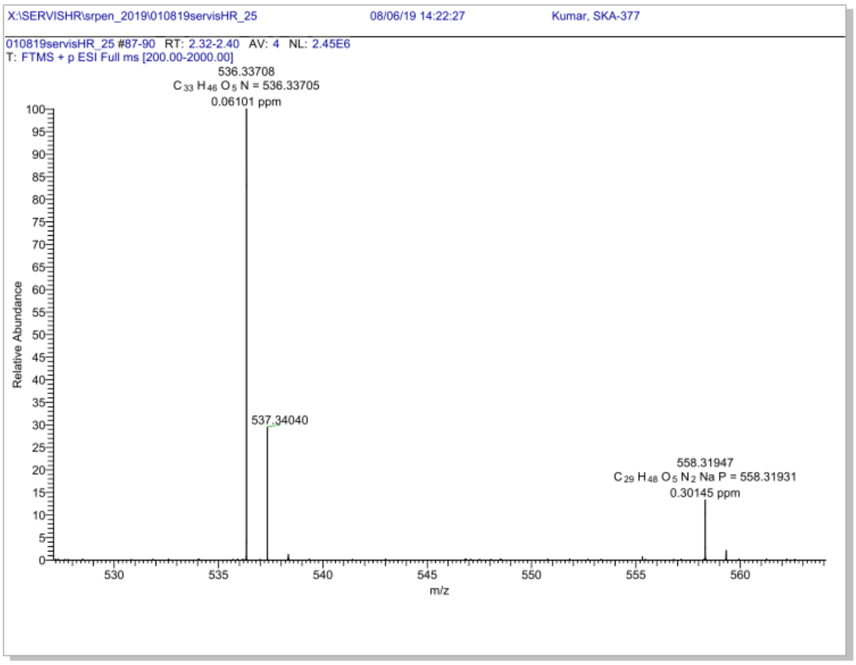

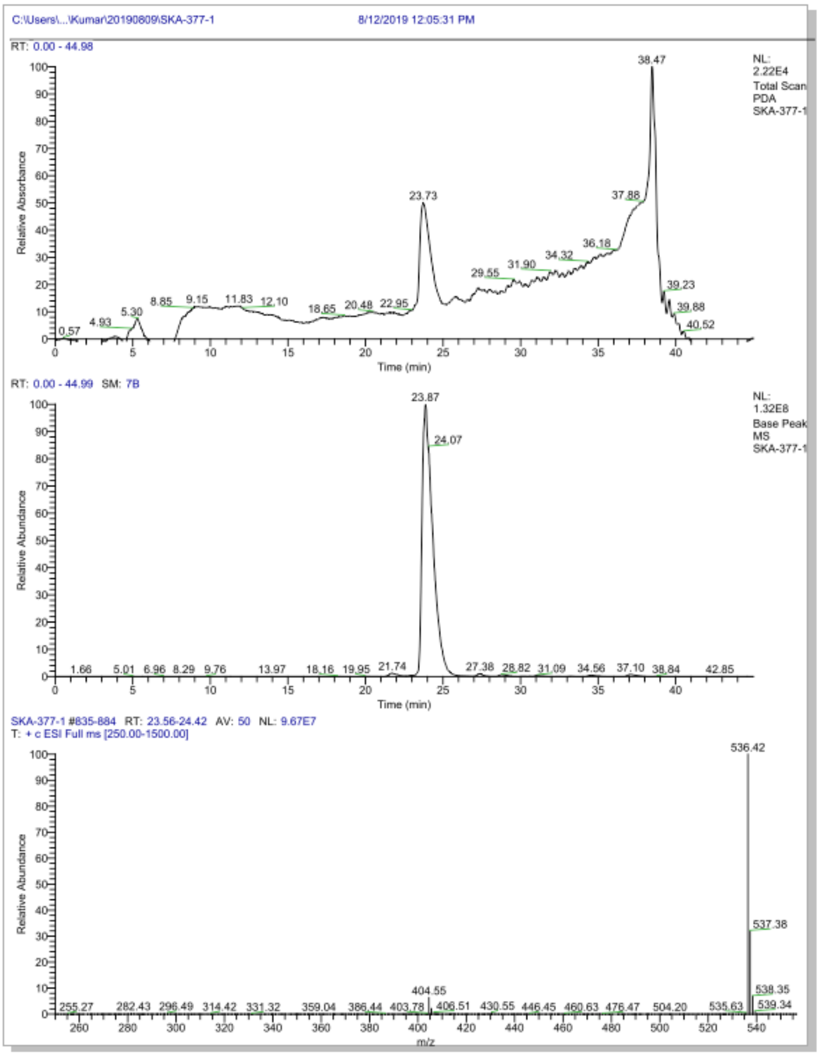

Figure S30. HR-MS and LC-MS spectra of compound **22**

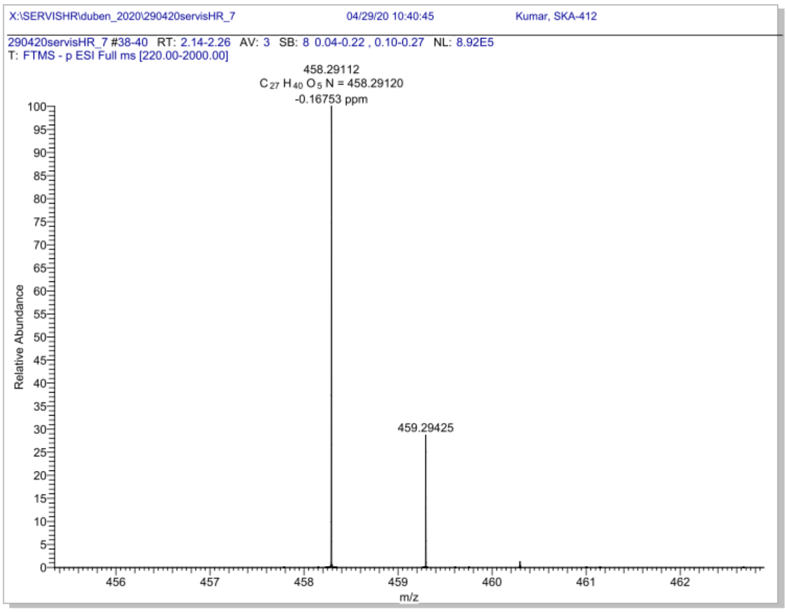

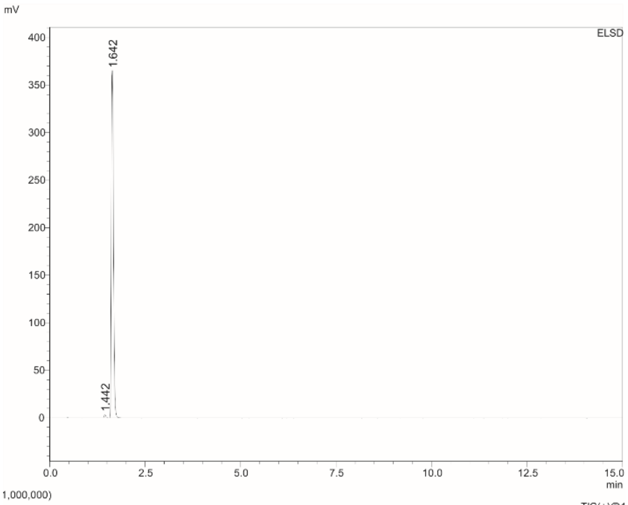

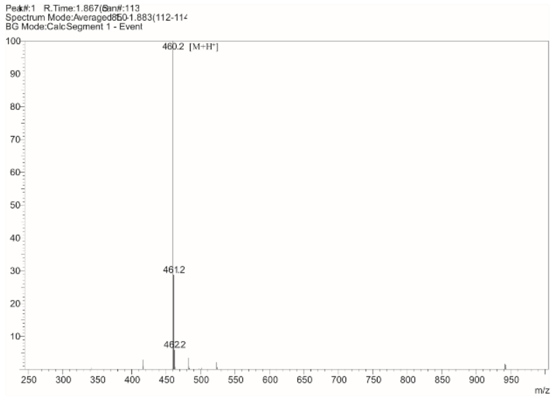

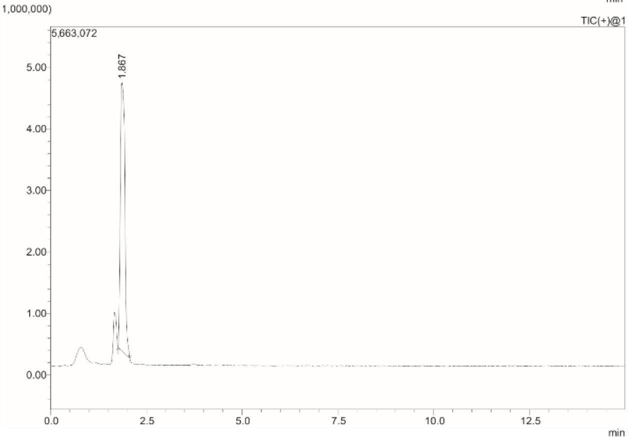

Box :
Position :
Method Filename : C8-80,20.lcm
Vial # : 1-4
Injection Volume : 0.3 uL
Date Acquired : 25-Aug-22 13:31:26

Comment : Isocratic, A: H2O, MeOH, FA (950:50:1); B: ACN; A:B (80:20), 0.5 mL/min
Column, Shim-pack Scepter, C8-120, 1.9 um, 2.1x100 mm, (Shimadzu)

ELSD

Ret. Time	Area	Area%
1.442	10867	0.718
1.642	1502855	99.282
	1513722	100.000

Figure S31. HR-MS and LC-MS spectra of compound **23**

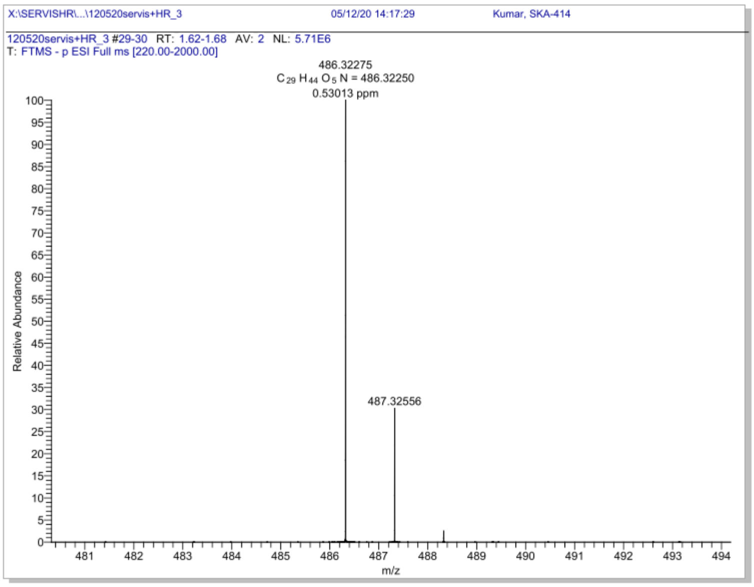

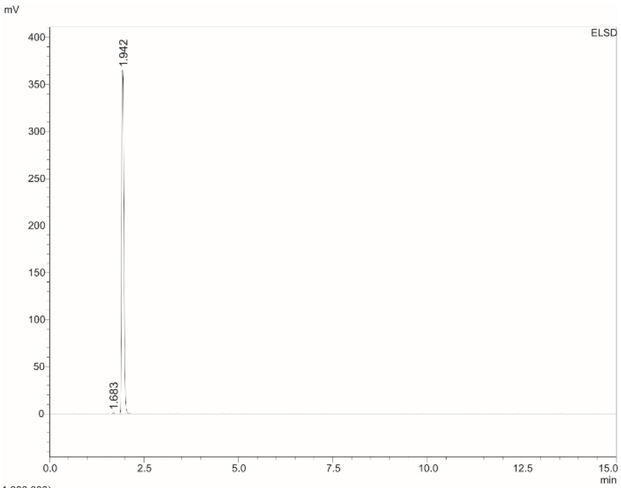

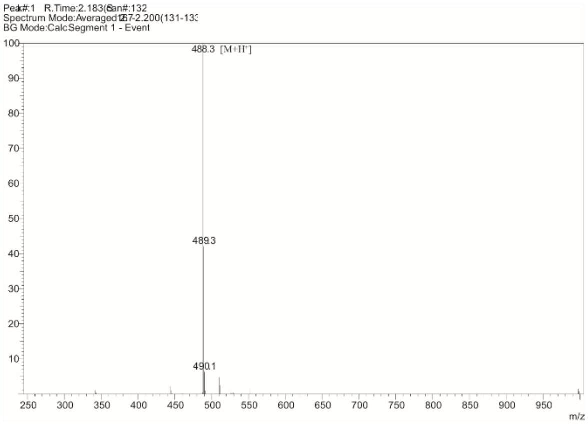

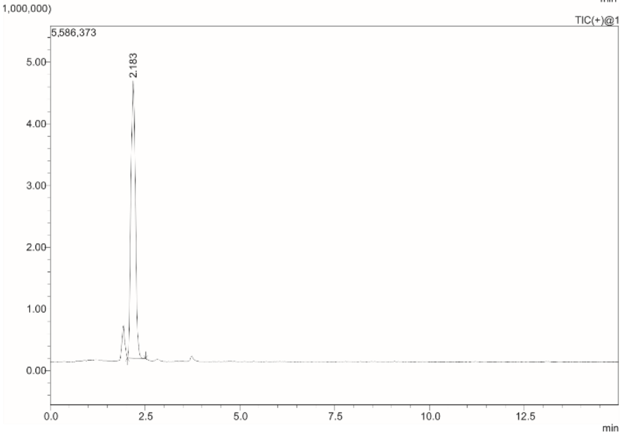

Box :
Position :
Method Filename : C8-80,20.lcm
Vial # : 1-5
Injection Volume : 0.3 uL
Date Acquired : 25-Aug-22 13:46:49

Comment : Isocratic, A: H2O, MeOH, FA (950:50:1); B: ACN; A:B (80:20), 0.5 mL/min
Column, Shim-pack Scepter, C8-120, 1.9 um, 2.1x100 mm, (Shimadzu)

ELSD

Ret. Time	Area	Area%
1.683	2353	0.157
1.942	1498508	99.843
	1500861	100.000

Figure S32. HR-MS and LC-MS spectra of compound **24**

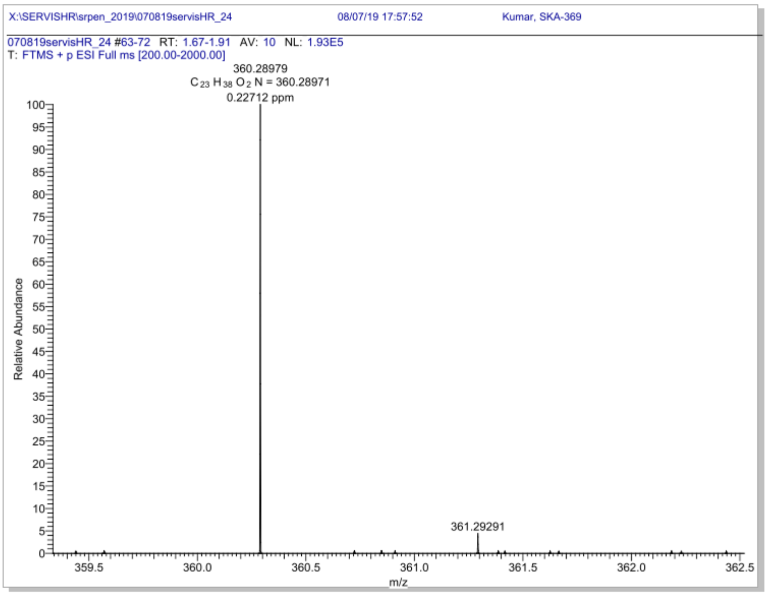

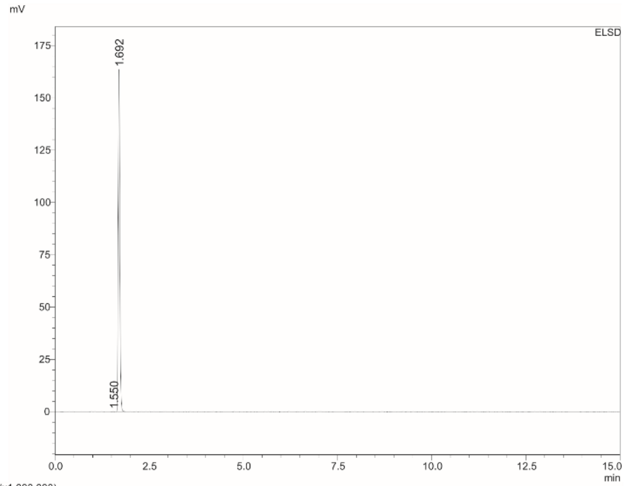

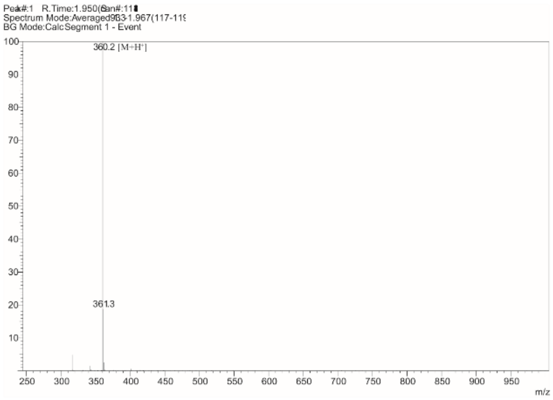

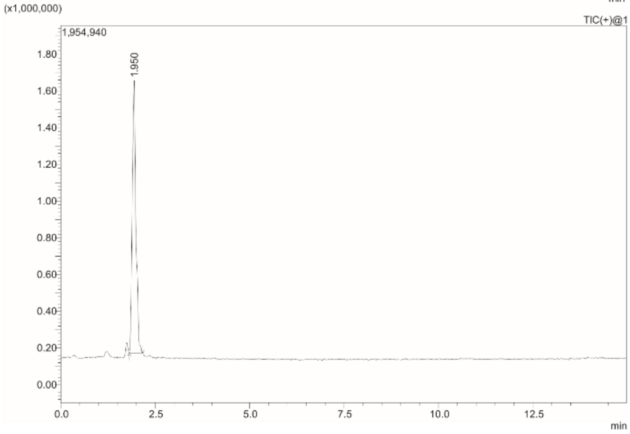

Box :
Position :
Method Filename : C8-80,20.lcm
Vial # : 1-1
Injection Volume : 0.4 uL
Date Acquired : 25-Aug-22 12:15:28

Comment : Isocratic, A: H2O, MeOH, FA (950:50:1); B: ACN; A:B (80:20), 0.5 mL/min Column, Shim-pack Scepter, C8-120, 1.9 um, 2.1x100 mm, (Shimadzu)

ELSD

Ret. Time	Area	Area%
1.550	215	0.044
1.692	493433	99.956
	493648	100.000

Figure S33. HR-MS and LC-MS spectra of compound **25**

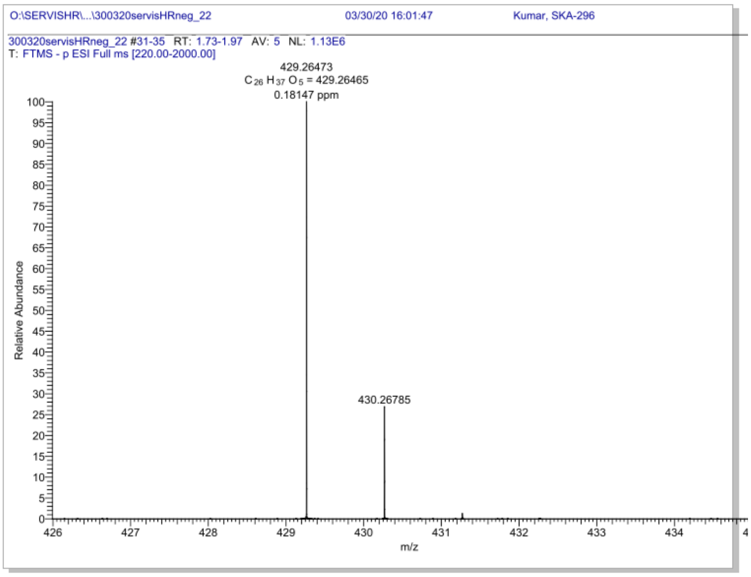

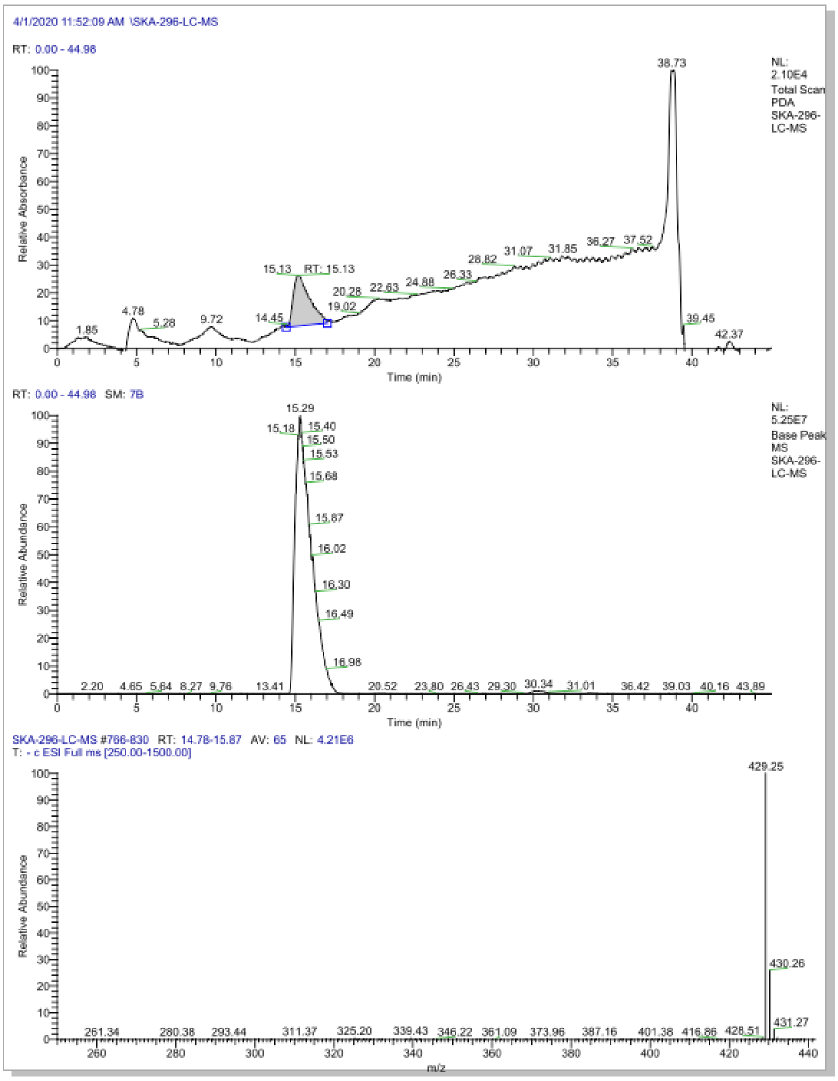

Figure S34. HR-MS and LC-MS spectra of compound **26**

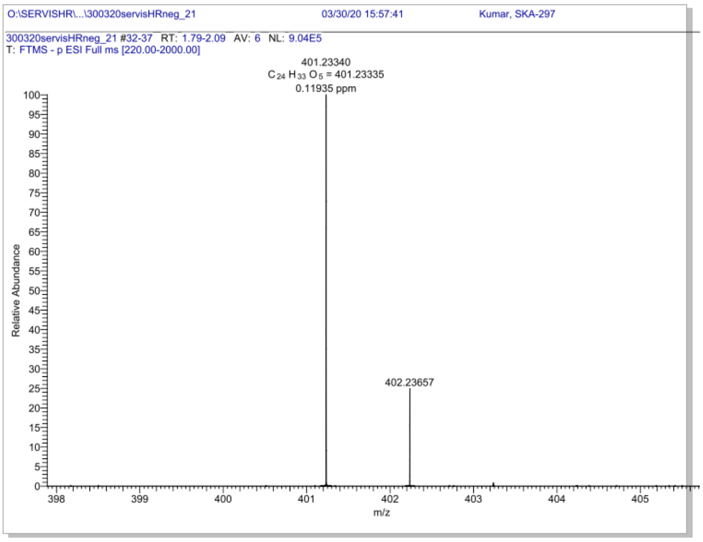

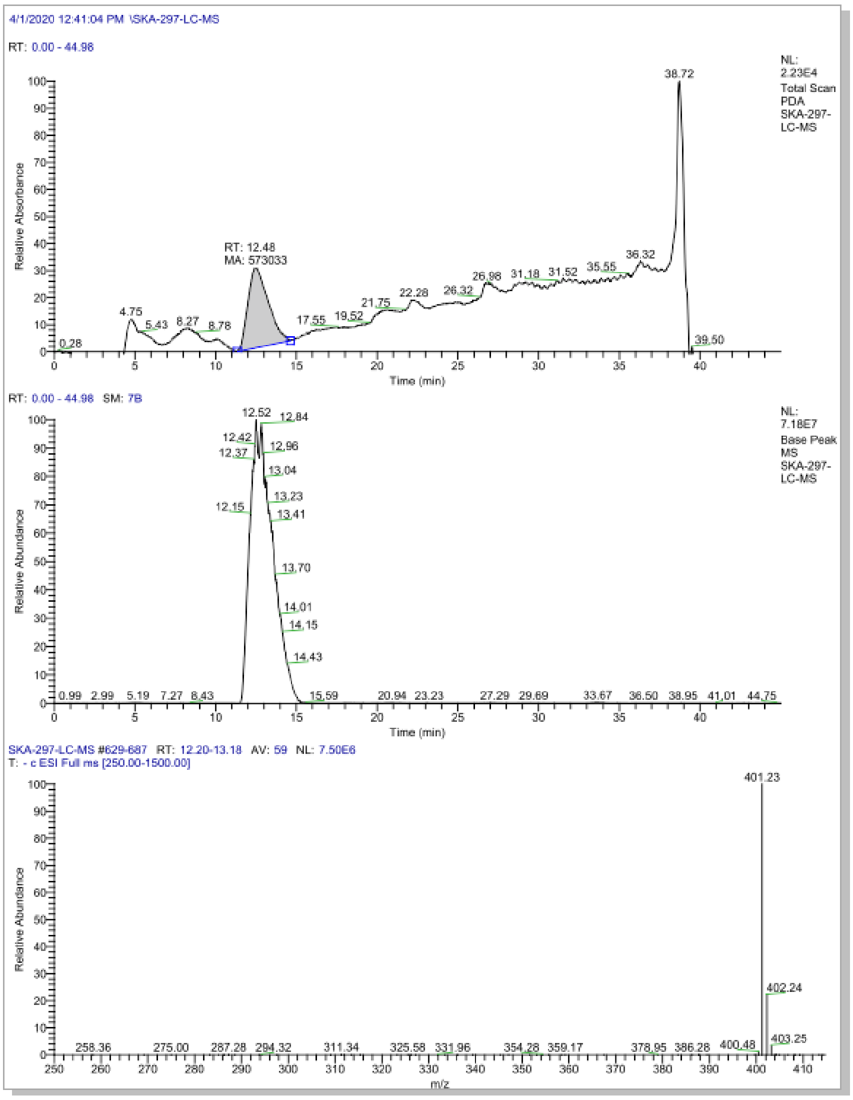

Figure S35. HR-MS and LC-MS spectra of compound **27**

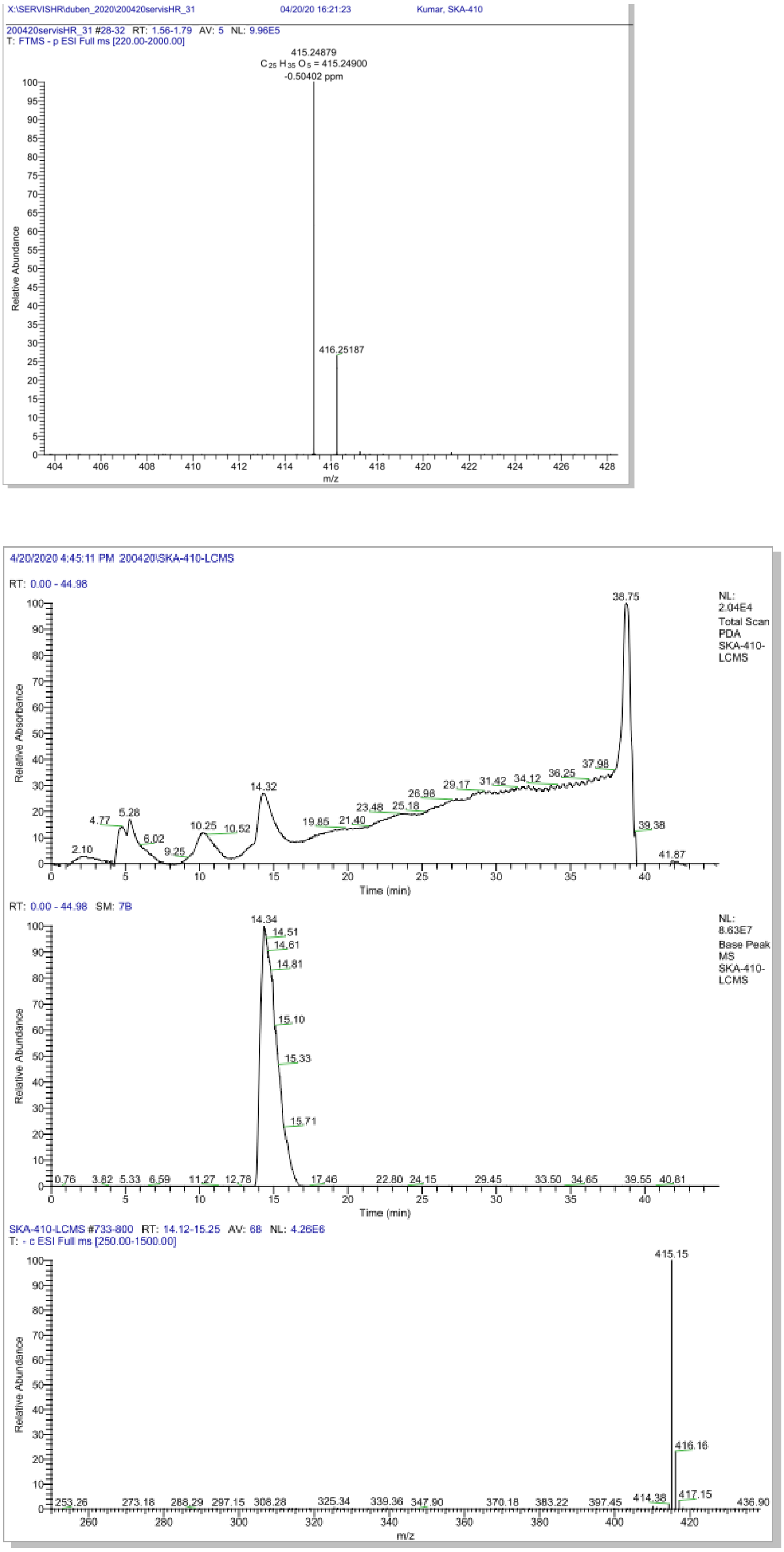

Figure S36. HR-MS and LC-MS spectra of compound **28**

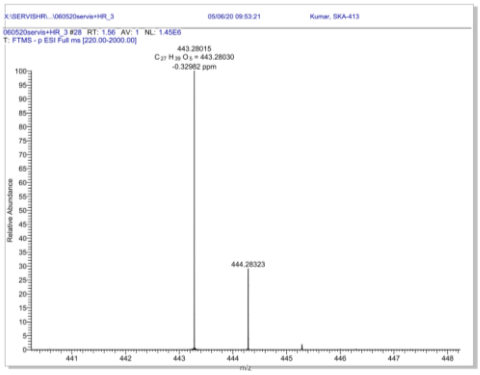

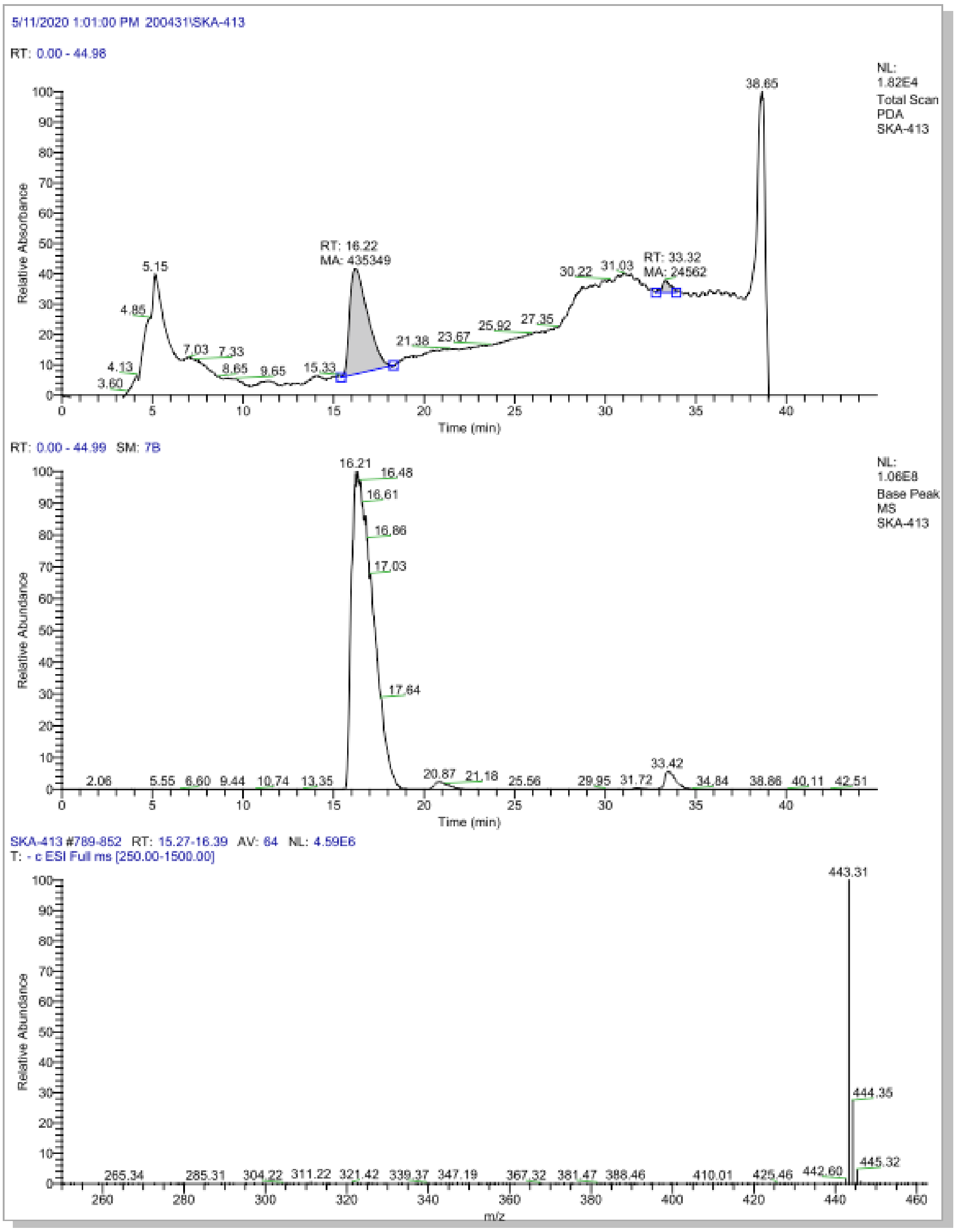

Supplement: RA-016-D5RA07716H-s001 [file RA-016-D5RA07716H-s001.pdf]
